# Computational investigation of thermal energy transfer enhancement in nanofluid in the presence of viscous dissipation, Joule heating and yield stress: a local similarity approach

**DOI:** 10.1038/s41598-026-64086-7

**Published:** 2026-08-08

**Authors:** M. Nawaz, M. Ahmed, Sayer Obaid Alharbi, A. S. Shflot

**Affiliations:** 1 Mathematics of Applied Mathematics and Statistics, Institute of Space Technology, P.O. Box 2750, Islamabad, 44000 Pakistan; 2 Mathematics Department, College of Science, Majmaah University, Al-Zulfi, Majmaah, 11952 Saudi Arabia; 3 Department of Mathematics, College of Sciences, King Khalid University, Abha, 61413 Saudi Arabia

**Keywords:** Darcy-Forchheimer porous medium, Yield stress, Thermal performance, Multi-nano-scale particles, Numerical study, Local Nusselt number, Energy science and technology, Engineering, Mathematics and computing, Nanoscience and technology, Physics

## Abstract

Thermal transport by fluids has numerous applications, including thermal radiators, thermal and cooling systems, MHD generators, etc. In this study, the thermal transport performance of the Casson fluid is investigated in response to the inclusion of three combinations of nanoscale particles. These combinations are: (i) $\:F{e}_{3}{O}_{4}$, (ii) $\:F{e}_{3}{O}_{4}$-* Co* and (iii) $\:F{e}_{3}{O}_{4}$-* Co*-* Ni*. These combinations are called mono-nanoparticles, di-nanoparticles, and tri-nanoparticles, respectively. Kerosene oil is used as the base fluid. The boundary layer approximations are used for the simplification of the system of governing PDEs under local thermal equilibrium. A local similarity transformation is used to reduce the governing equations to a system of ODEs. Numerical solutions of the transformed system of BVPs using a numerical method called BVP4C. The optimization in thermal enhancement is aimed at increasing the thermal conductivity due to the dispersion of multi-nanoscale particles. A porous medium creates a resistance to the flow, which reduces the convective heat transfer. Consequently, the heat transport rate decreases. Eventually, the local Nusselt number decreases. Thus, flow in practical applications should not be in the porous medium where the local Nusselt number needs to be optimized. The viscoplasticity reduces the skin friction coefficient. Moreover, the boundary layer thickness associated with tri-nanofluid ($\:F{e}_{3}{O}_{4}$-* Co*-* Ni*-kerosene oil) is wider than that with $\:F{e}_{3}{O}_{4}$-* Co*-kerosene oil and $\:F{e}_{3}{O}_{4}$-Kerosene oil. It is established in this study that kerosene containing $\:F{e}_{3}{O}_{4},\:Co$ and * Ni* has the highest effective thermal conductivity in comparison with kerosene oil, having $\:F{e}_{3}{O}_{4}\:$and * Co* and kerosene oil with $\:F{e}_{3}{O}_{4}$. Therefore, it is concluded that among$\:\:F{e}_{3}{O}_{4}$-* Co*-* Ni*-kerosene oil, $\:F{e}_{3}{O}_{4}$-* Co*-kerosene oil and $\:F{e}_{3}{O}_{4}$-kerosene oil, $\:F{e}_{3}{O}_{4}$-* Co*-* Ni*-kerosene oil is the best working fluid concerning heat transport. The thermal radiations are electromagnetic waves that carry heat energy with them, and therefore, the thermal boundary layer thickness is reduced, and the local Nusselt number is increased with an increase in the thermal radiations.

## Introduction

 Fluids are frequently used to transport heat and mass. The quantity of heat and mass transported is directly proportional to diffusion coefficients (thermal conductivity and mass diffusion coefficient). Unfortunately, the fluids do not have thermal conductivity and mass diffusion coefficients as high as required for the optimal transport of mass and heat. To fulfil this need, several techniques have been introduced and utilized. In this regard, both active and passive techniques have been used. Advancements in nanotechnology and the invention of methods and principles for the preparation of nanoparticles have brought a revolution in almost all fields, including thermal and mass transport systems. As researchers and scientists always strive to introduce innovative ideas, concepts, and techniques, they started investigating the role of nanoparticles in enhancing thermal conductivity and mass diffusivity. Now, it is a verified fact that thermal improvement by increasing thermal conductivity is possible through the dispersion of nanoparticles. Because of the usage of nanoparticles, researchers have published extensive research related to the applications, usage, and advantages of nanoparticles and nanofluids. However, in the context of this study, we are describing the role of nanoparticles in heat and mass transfer. For example, Alsenafi et al.^1^ studied the combined effects of thermal slip, chemical reaction, and porous media on convective transport of heat and mass. Non-Fourier heat and mass transport were numerically studied by Alsenafi et al.^2^ using the Galerkin finite element method. Alsenafi et al.^3^ numerically investigated the influence of heat transfer in fluid in the presence of a magnetic field. Madkhali et al.^4^ published a study on the enhancement of heat transport in a fluid subjected to Brownian motion and thermophoresis effects. They concluded that hybrid nanoparticles are more effective in raising thermal conductivity in comparison with single types of nanoparticles. Farooq et al.^5^ published a study on the role of nanoparticles in enhancing the thermal conductivity of the fluid used for transporting heat. Shamshuddin et al.^6^ investigated the influence of hybrid nanoparticles on Thermal enhancement in fluid over a bio convectively heated surface and they concluded that hybrid nanoparticles are more effective in raising thermal conductivity of the fluid in comparison of mono-nanoparticles. Rosca et al.^7^ studied an optimization in thermal transport of fluid radiating thermal radiation. They explored how hybrid nanoparticles and thermal radiation simultaneously affect the thermal performance of the fluid subjected to the permeability of the surface. Waini et al.^8^ explores an increase in the capability of water to transport heat efficiently when $\:A{l}_{2}{O}_{3}$ nanoparticles are homogeneously mixed in water. Hussain et al.^9^ and Jayaprakash et al.^10^ also examined the role of hybrid nanoparticles in enhancing the thermal performance of the fluid over a moving heated surface. For more details, see references^11–15^.

Non-Newtonian fluids are those that do not obey Newton’s law of viscosity. Many fluids prepared at the industrial level are non-Newtonian and vary in their rheology. This diversity in the rheological characteristics of fluids puts a requirement on diverse rheological models (stress-strain relations) to study the movement of fluids and related mechanisms accurately. Having this in mind, researchers have proposed several stress-strain relationships. The Casson fluid is a non-Newtonian fluid and exhibits yield stress properties. The stress-strain relation^16^ exhibiting yield stress is written below.

1 $$\:{\boldsymbol{\tau\:}}_{\boldsymbol{i}\boldsymbol{j}}=\left\{\genfrac{}{}{0pt}{}{\begin{array}{c}2{\left({\mu\:}_{B}+\frac{{p}_{y}}{\sqrt{{2\pi\:}}}\right)e}_{ij},\:\:\:\pi\:>{\pi\:}_{c}\\\:\end{array}}{2{\left({\mu\:}_{B}+\frac{{p}_{y}}{\sqrt{{2\pi\:}_{c}}}\right)e}_{ij},\:\:\:{\pi\:}_{c}>\pi\:}\right.$$

where $\:{\mu\:}_{B}$, $\:{e}_{ij}$, $\:{\pi\:}_{c}$, and $\:{p}_{y}$, are the plastic dynamic viscosity rate of deformation, critical value of $\:\pi\:$, and the yield stress, respectively. The stress-strain relation in Eq. (1) has been used in several studies. Here, we describe some of the latest studies. For instance, Abideen and Saif^17^ model the heat transport mechanism in Casson containing carbon nanotubes and derived numerical solutions of the problems to simulate the features of heat transport under the change of related parameters. They also analysed the influence of yield stress on heat transport enhancement. Alkasasbeh^18^ studied mixed convective heat transport in Casson fluid subjected to a magnetic field. Abbasi et al.^19^ analysed the impact of thermal radiation and binary chemical reaction on mixed convective transport in Casson fluid in the presence of activation energy. Saeed et al.^20^ numerically analysed the influence of variable thermal conductivity on heat transfer in a material exhibiting yield stress. Jalili et al.^21^ discussed the impact of nonlinear thermal radiation and porous medium on heat transport in a liquid exhibiting yield stress. Mohanty et al.^22^ studied the combined effects of yield stress, thermal relaxation time, and porous medium on convective heat transfer in the viscoplastic fluid using the Casson constitutive model. Memon et al.^23^ studied the combined effects of an inclined magnetic field and a porous medium on the flow of Casson fluid between two squeezing plates. Das et al.^24^ discussed the combined effects of Hall current, chemical reaction, and thermal radiation on convective transport of heat and mass in Casson fluid. Shehzad et al.^25^ discuss three-dimensional convective transfer in Casson fluid, the presence of a magnetic field, and heat generation. However, it is important to note that the effects of mono-, di-, and tri-nanoparticles on heat transfer in free dimensional flow of Casson fluid in porous medium have not been studied yet.

Fluid flow in a porous medium occurs in numerous applications. Drilling out of oil from soil, petroleum engineering, chemical reactions, and soil sciences are areas where fluid flow in porous media is encountered. The resistance faced by fluid flow due to the presence of a porous medium is a force that opposes the flow. The magnitude of this resistive force depends on the permeability of the porous medium, fluid viscosity, and the length of the porous medium. Therefore, more than one model for the magnitude of the porous medium’s force on fluid flow has been derived. In this regard, Henry Darcy proved and found that the resistance force offered by porous media to oppose the flow is directly and linearly proportional to the velocity of the fluid. Later, it was found that some porous media do not obey Darcy’s law. In such a porous medium, force has a nonlinear relationship with the fluid. The well-known law is the non-Darcy law, which is called the Forchheimer-Darcy law. The Forchheimer-Darcy model has been used in various studies. For example, Hafez et al.^26^ investigated the impact of the Darcy-Forchheimer porous medium and electroosmosis on heat transfer in Casson fluid. Nasir et al.^27^ performed simulations related to the combined effects of chemical reaction, porous medium, and convective boundary conditions on heat transfer in a fluid exhibiting yield stress. For more insights, please see references^28–34^. However, it is important to note that the present study is on three-dimensional heat transfer in Casson fluid under nonlinear thermal radiation.

Electrically conducting fluids can conduct an electric current. However, due to particle resistance, some of the electrical energy changes to heat energy. This phenomenon of change of electrical energy into heat energy is called ohmic dissipation or Joule heating. Like solids, ohmic dissipation is also observed in fluids. The heat energy produced due to ohmic dissipation becomes part of the energy of the fluid and, therefore, must be taken into account for the energy balance equation. It is also important to mention that in MHD flows, the current is produced due to a change in magnetic flux due to the movement of fluid across the magnetic lines. In the considered fluid flow situation, the magnetic field is applied, and the fluid moves due to the wall movement. Hence, current produces part of which changes to heat energy, which must be taken into the energy balance equation. This gives additional terms in the energy equation, which is a challenge to the computation procedure. However, by accepting this challenge, this effect is taken into account. The Joule heating phenomenon has been analysed and discussed in several studies. For instance, Nasir et al.^35^ published a computational study on the influence of the porous medium, Joule heating, magnetic field, and yield stress on triple transport in Casson fluid. It is very important to mention that no study on the impact of joule heating in three-dimensional flow of Casson fluid containing mono-, di-, and tri-nanoparticles so far. This aspect of fluid flow and heat transfer in Casson fluid subjected to dispersion of nanoparticles, Joule heating, and porous medium is being explored in the present investigation. Shamshuddin et al.^36^ analysed the influence of Joule heating on mixed convective heat transfer in a fluid porous medium. Goyal and Srinivas^37^ analysed the impact of Joule heating and the magnetic field, and the chemical reaction of entropy generation. Sreenivasulu et al.^38^ examine the simultaneous impact of Joule heating and a magnetic field on heat transport in Casson fluid flowing over a movable surface.

It is a fact that there are fluids that emit thermal radiation when they undergo thermal changes. Thermal radiation has the characteristic of carrying heat energy with it. This escape of heat energy results in a decrease in temperature. Consequently, fluid cools down, and its ability to transport heat increases. This means that thermally radiating fluids are good in the context of working fluids used for cooling purposes. Given this relevance, thermal radiation in fluids has been studied extensively. For example, Zainal et al.^39^ discussed the effect of thermal radiation on heat transfer in a hybrid nanofluid over a permeable moving surface in the presence of thermal radiation. Gajbhiye et al.^40^ studied thermal radiation and chemical reaction on the transport mechanism in Casson fluid. Dandu et al.^41^ studied thermal radiation effects on heat transfer in a fluid subjected to diffusion-thermo and activation energy in MHD flow of Casson fluid. Wang et al.^42^ numerically analyse the impact of thermal radiation, heat generation, and Dispersion of nanoparticles on heat transfer in Casson fluid. Thermal radiation is also studied in references^43–46^.

A thorough analysis of the comparative thermal enhancement properties of mono-nanoparticle, hybrid (di-nanoparticle), and ternary (tri-nanoparticle) suspensions in a Casson fluid has not yet been published, despite notable advancements in the study of Casson fluid flow and heat transfer. Specifically, no research has thoroughly investigated how these nanoparticle configurations affect the thermal performance of Casson fluid flow caused by a two-dimensional moving wall while concurrently taking nonlinear thermal radiation effects, internal heat generation, and Joule heating into account. Therefore, by creating and evaluating a novel mathematical model that permits a thorough evaluation of the relative heat transfer enhancement attained through mono-, di-, and tri-nanoparticle dispersions under the combined action of these physically significant thermal mechanisms, the current work closes this significant research gap. This study is organized into five sections. Section  1 is for background and a literature review. Section  2 contains the problem design and its formulation. The numerical scheme is implemented in Sect.  3. The outcomes are discussed and explained in Sect.  4. The key findings are listed in Sect.  5.

## Problem statement and formulation

**Fig. 1:**
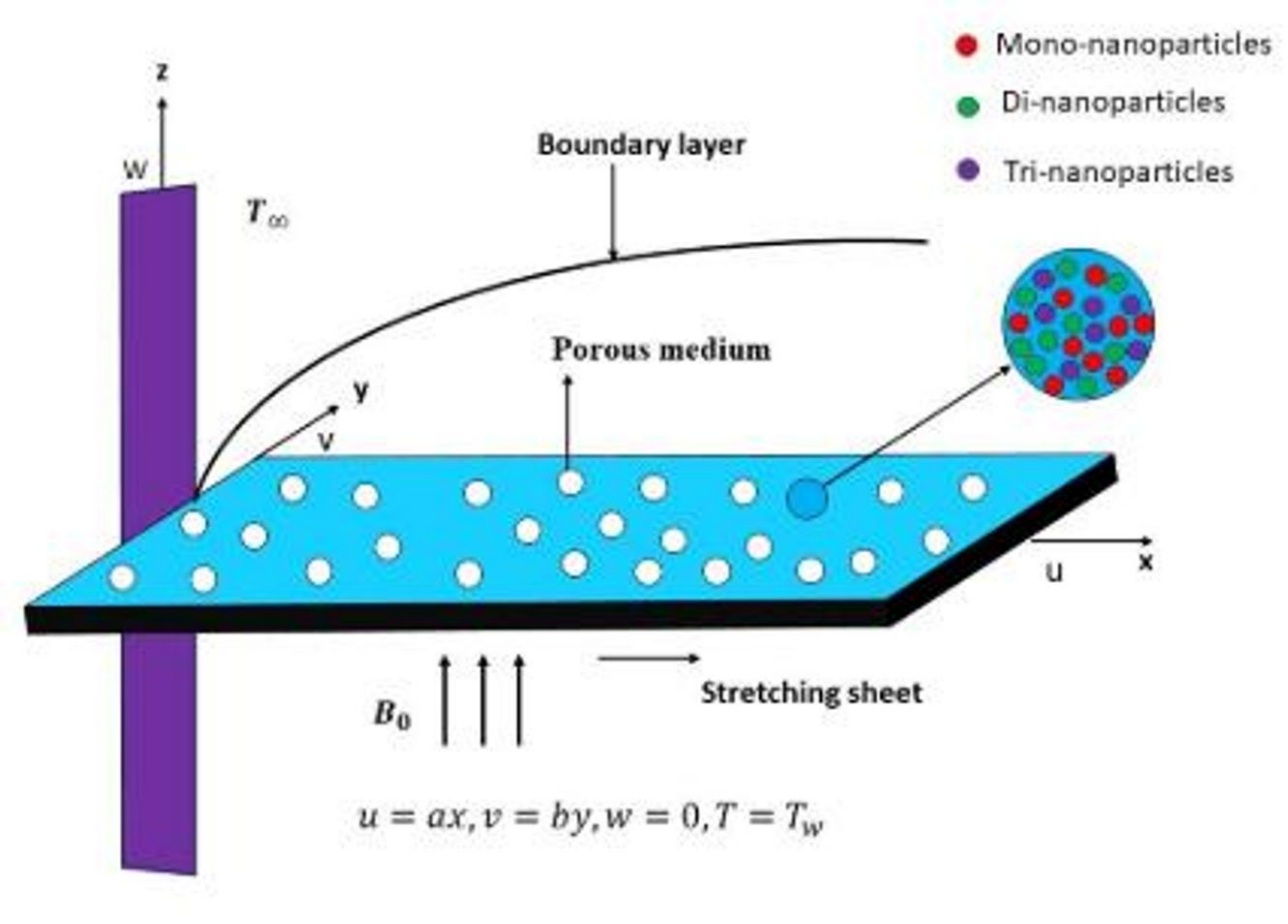
Flow configuration in the Cartesian coordinate system.

Let us consider the Casson fluid existing over a two-dimensional moving heated surface subjected to a magnetic field. The surface is heated and has a constant temperature. A two-dimensional moving surface causes a three-dimensional flow of Casson fluid. This flow is responsible for the mixed convective transport of heat. The thermal performance of Casson fluids is expected to increase with the dispersion of mono-, di-, and tri-nanoscale particles. The fluid flow and heat transport in a Casson fluid in a porous medium obey the Darcy-Forchheimer model of resistive force. The flow configuration is given in Fig. 1. The above-stated situation is modeled by imposing the following assumptions:

i. In the present analysis, the Local thermal equilibrium assumption is adopted, implying that the temperatures of the nanofluid and porous matrix are identical ($\:T={T}_{s}$, $\:{T}_{s}$ is the temperature of the porous matrix). This assumption is justified by the rapid interfacial heat exchange between the two phases, which renders the temperature difference negligible. Consequently, a single energy equation adequately describes the heat transfer in a fluid in a porous medium. For this case, an additional heat conduction equation for heat conduction in the porous matrix is not required.
ii. Instead of the Brinkman-extended Darcy-Forchheimer model^47^, the Darcy–Forchheimer porous media model is assumed to be valid.
iii. Flow is incompressible.
iv. The surface has a constant temperature.
v. Casson fluid is heat-generating/heat-absorbing.
vi. A magnetic field of constant strength is imposed along the * z-*axis.
vii. Flow in a porous medium, which offers a resistive force to fluid flow. The Darcy-Forchheimer porous-medium model quantifies this resistive force.
viii. Ohmic dissipation is considered.
ix. The flow of fluid develops a boundary layer, and, therefore, boundary layer approximations are applied to the full conservation equations to get their simplified forms.

### Limitations of this study

The results are valid only under the assumptions stated above. By relaxing the above-stated assumptions, the results are not valid. Further, the findings of the present study are limited to the case when the fluid and porous medium matrix have the same temperature. Moreover, the results are not the same as those obtained by the Brinkman-extended Darcy-Forchheimer model^47^.

### Assumptions and their justification

The study is conducted under certain assumptions stated above. These are the realistic assumptions that correspond to several real-life applications. The justification is stated below.

- In this case, the density of the fluid remains constant, and no volumetric compression occurs. This condition does not hold in general. However, there may be several fluid situations where the incompressibility condition is valid, especially for the case of the flow of liquids. As in the present case, Casson fluid is an incompressible fluid. Therefore, incompressibility is considered and is justified.
- There are several man-made processes where the wall temperature remains constant per the requirements of the process. Therefore, constant wall temperature is taken in this study.
- Variable and constant magnetic fields are usually encountered in several related applications. However, a constant applied magnetic field is taken into account. It means the findings are valid for those practical processes where a constant magnetic field exists.
- Several industrial and engineering applications are related to flow in porous media (the applications are listed in Sect.  1). Therefore, flow in porous media has significance.
- Almost all the fluids are electrically conducting, and ohmic dissipation occurs when the fluid is electrically conducting. Therefore, heat generated by ohmic dissipation cannot be ignored. Otherwise, analysis leads to erroneous results.
- It is a proven fact (see studies discussed in Sect.  1 as literature review) that fluids induce heat when they pass through thermal changes. This is called the hate generation and is also considered in this study.

It is important to mention that in moderate- to high-velocity flows in porous media, the Forchheimer inertia effect is significant, especially when the pore Reynolds number ($\:R{e}_{p}\gtrsim\:1$) is greater than unity, making inertial forces equal to or greater than viscous forces. Darcy’s linear model is insufficient in these circumstances, and the nonlinear Forchheimer term needs to be included.

The set of PDEs by using the boundary layer approximation, is given below^25^

2 $$\:\frac{\partial\:u}{\partial\:x}+\frac{\partial\:v}{\partial\:y}+\frac{\partial\:w}{\partial\:z}=0,$$

3 $$\:u\frac{\partial\:u}{\partial\:x}+v\frac{\partial\:u}{\partial\:y}+w\frac{\partial\:u}{\partial\:z}={\nu\:}_{tnf}\left(1+\frac{1}{\beta\:}\right)\left(\frac{{\partial\:}^{2}u}{\partial\:{z}^{2}}\right)-\frac{{\sigma\:}_{tnf}}{{\rho\:}_{tnf}}{B}_{0}^{2}u-\frac{{\nu\:}_{tnf}}{{k}_{p}}u-\frac{{C}_{b}}{x\sqrt{{k}_{p}}}{u}^{2},$$

4 $$\:u\frac{\partial\:v}{\partial\:x}+v\frac{\partial\:v}{\partial\:y}+w\frac{\partial\:v}{\partial\:z}=\:{\nu\:}_{tnf}\left(1+\frac{1}{\beta\:}\right)\left(\frac{{\partial\:}^{2}v}{\partial\:{z}^{2}}\right)-\frac{{\sigma\:}_{tnf}}{{\rho\:}_{tnf}}{B}_{0}^{2}v-\frac{{\nu\:}_{tnf}}{{k}_{p}}v-\frac{{C}_{b}}{y\sqrt{{k}_{p}}}{v}^{2},$$

$$\:u\frac{\partial\:T}{\partial\:x}+v\frac{\partial\:T}{\partial\:y}+w\frac{\partial\:T}{\partial\:z}=\frac{{K}_{tnf}}{(\rho\:{c}_{p}{)}_{tnf}}\left(\frac{{\partial\:}^{2}T}{\partial\:{z}^{2}}\right)+\frac{Q}{(\rho\:{c}_{p}{)}_{tnf}}\left(T-{T}_{\infty\:}\right)+\frac{{\sigma\:}_{tnf}{B}_{0}^{2}}{(\rho\:{c}_{p}{)}_{tnf}}\:\left({u}^{2}+{v}^{2}\right)$$

5 $$\:+\frac{16\:{\sigma\:}^{*}}{3(\rho\:{c}_{p}{)}_{tnf}{k}^{*}}\:\frac{\partial\:}{\partial\:z}\left({T}^{3}\frac{\partial\:T}{\partial\:z}\right),$$

The boundary conditions followed by the no-slip assumption are:

6 $$\:\left.\begin{array}{c}u\left(0\right)={U}_{w}\left(x\right)=ax,\:v\left(0\right)={V}_{w}\left(y\right)=by,\:w\left(0\right)=0,\:T\left(0\right)={T}_{w},\\\:u\left(\infty\:\right)\to\:0,\:v\left(\infty\:\right)\to\:0,\:T\left(\infty\:\right){\to\:T}_{\infty\:}.\end{array}\right\}$$

where $\:\left[\:u,\:v,\:w\right]$, * T*, * Q*, $\:{K}_{tnf}$, $\:({c}_{p}{)}_{tnf}$, $\:{\rho\:}_{tnf}$, $\:{\sigma\:}_{tnf}$, $\:{k}^{*}$, $\:{B}_{0}$, $\:{\nu\:}_{tnf}$, $\:\beta\:,$ and $\:{k}_{p}$, respectively, are velocity components of fluid, temperature, heat generation/absorption coefficient, thermal conductivity, specific heat, density, electrical conductivity, mean absorption coefficient, magnetic induction, fluid kinematic viscosity, Casson fluid parameter, and permeability of porous medium.

To transform Eqs. (3)-(6) into their dimensionless form, the following transformations is used.

7 $$\:u=ax{f}^{{\prime\:}}\left(\eta\:\right),\:\:\:v=ay{g}^{{\prime\:}}\left(\eta\:\right),\:w=-\sqrt{a{\nu\:}_{f}}\left(f(\eta\:\right)+g\left(\eta\:\right)),\theta\:(\eta\:)=\:\frac{T-{T}_{\infty\:}}{{T}_{w}-{T}_{\infty\:}},\:\:\:\eta\:=z\sqrt{\frac{a}{\nu\:_{f}}}$$

Hence, on gets

8 $$\:\frac{{A}_{1}}{{A}_{2}}\left(1+\frac{1}{\beta\:}\right){f}^{{\prime\:}{\prime\:}{\prime\:}}+\left[f+g\right]{f}^{{\prime\:}{\prime\:}}-[1+Fr]{{f}^{{\prime\:}}}^{2}-\left(\frac{{A}_{3}}{{A}_{2}}{M}^{2}+\frac{{A}_{1}}{{A}_{2}}\lambda\:\right){f}^{{\prime\:}}=0,$$

9 $$\:\frac{{A}_{1}}{{A}_{2}}\left(1+\frac{1}{\beta\:}\right){g}^{{\prime\:}{\prime\:}{\prime\:}}+\left[f+g\right]{g}^{{\prime\:}{\prime\:}}-[1+Fr]{{g}^{{\prime\:}}}^{2}-\left(\frac{{A}_{3}}{{A}_{2}}{M}^{2}+\frac{{A}_{1}}{{A}_{2}}\lambda\:\right){g}^{{\prime\:}}=0,$$

$$\:\frac{{A}_{4}}{{A}_{5}}{\theta\:}^{{\prime\:}{\prime\:}}+\mathrm{P}\mathrm{r}\left[f+g\right]{\theta\:}^{{\prime\:}}+\frac{\mathrm{P}\mathrm{r}B}{{A}_{5}}\theta\:+\frac{{A}_{3}}{{A}_{5}}\mathrm{P}\mathrm{r}{M}^{2}\left({Ec}_{x}{{f}^{{\prime\:}}}^{2}+{Ec}_{y}{{g}^{{\prime\:}}}^{2}\right)+$$

10 $$\:\frac{4}{3{A}_{5}Nr}\frac{d}{d\eta\:}\left[\right(1+\left({\theta\:}_{w}-1\right)\theta\:{)}^{3}{\theta\:}^{{\prime\:}}]=0,$$

11 $$\:\left.\begin{array}{c}f\left(0\right)=0,\:g\left(0\right)=0,\:{f}^{{\prime\:}}\left(0\right)=1,\:{g}^{{\prime\:}}\left(0\right)=\alpha\:,\:\theta\:\left(0\right)=1,\\\:{f}^{{\prime\:}}\left(\infty\:\right)\to\:0,g^{\prime\:}\left(\infty\:\right)\to\:0,\:\theta\:\left(\infty\:\right)\to\:0.\end{array}\right\},$$

where $\:{A}_{1}=\frac{{\mu\:}_{tnf}}{{\mu\:}_{f}}$, $\:{A}_{2}=\frac{{\rho\:}_{tnf}}{{\rho\:}_{f}}$, $\:{A}_{3}=\frac{{\sigma\:}_{tnf}}{{\sigma\:}_{f}}$, $\:{A}_{4}=\frac{{K}_{tnf}}{{K}_{f}}$, $\:{A}_{5}=\frac{{\left(\rho\:{c}_{p}\right)}_{tnf}}{{\left(\rho\:{c}_{p}\right)}_{f}}$, $\:\beta\:=\frac{{\mu\:}_{\boldsymbol{B}}\sqrt{{2\pi\:}_{c}}}{{p}_{y}}$ is the Casson fluid parameter, $\:{M}^{2}=\frac{{\sigma\:}_{f}{B}_{0}^{2}}{a{\rho\:}_{f}}\:$is the Hartman number, $\:\lambda\:=\frac{{k}_{p}a}{{\nu\:}_{f}}\:$is the Darcy porous medium parameter, $\:\mathrm{P}\mathrm{r}=\frac{{\left(\mu\:{c}_{p}\right)}_{f}}{{K}_{f}}$ is the Prandtl number, $\:{\theta\:}_{w}=\:\frac{{T}_{w}}{{T}_{\infty\:}}>1$ is the temperature ratio parameter, $\:\alpha\:=\frac{b}{a}$ is the ratio parameter, $\:{\left(Ec\right)}_{x}=\frac{{a}^{2}{x}^{2}}{({c}_{p}{)}_{f}\left({T}_{w}-{T}_{\infty\:}\right)}\:$and $\:(Ec{)}_{y}=\frac{{a}^{2}{y}^{2}}{({c}_{p}{)}_{f}\left({T}_{w}-{T}_{\infty\:}\right)}$ are the local Eckert numbers, $\:B=\frac{Q}{a{\left(\rho\:{c}_{p}\right)}_{f}}$ is the heat generation or absorption parameter, $\:Fr=\frac{{C}_{b}}{\sqrt{{k}_{p}}}$ is the Forchheimer number and $\:Nr=\:\frac{{K}_{f}{k}^{*}}{4{\sigma\:}^{*}{T}_{\infty\:}^{3\:}}\:$is the thermal radiation parameter.

It is worth mentioning that the current analysis adheres to the widely used local similarity approximation that is frequently used in stretching-sheet flow boundary-layer analyses. This method treats the dimensionless parameters, such as the Eckert numbers as locally constant and formulates the governing equations at a fixed streamwise point. As a result, rather than in the strict global sense, the altered equations continue to be self-similar within the local boundary-layer framework.

If $\:{C}_{fx}$ and $\:{C}_{fy}$ are the skin friction coefficients and $\:{Nu}_{x}$ is the local Nusselt number, then we have

12 $$\:{C}_{fx}=\frac{{\tau\:}_{wx}{|}_{z=0}}{\rho\:_{f}{u}_{w}^{2}},\:{C}_{fy}=\frac{{\tau\:}_{wy}{|}_{z=0}}{\rho\:_{f}{u}_{w}^{2}},\:{Nu}_{x}=\frac{x{q}_{w}{|}_{z=0}}{{K}_{f}({T}_{w}-{T}_{\infty\:})}\:,$$

where $\:{\tau\:}_{wx}{|}_{z=0}={\left[{\mu\:}_{tnf}\left(1+\frac{1}{\beta\:}\right)\frac{\partial\:u}{\partial\:z}\right]}_{z=0}$, $\:{\tau\:}_{wy}{|}_{z=0}={\left[{\mu\:}_{tnf}\left(1+\frac{1}{\beta\:}\right)\frac{\partial\:v}{\partial\:z}\right]}_{z=0}$, are the wall shear stresses and $\:{q}_{w}={\left[{-K}_{tnf}\left(\frac{\partial\:T}{\partial\:Z}\right)+{q}_{r}\right]}_{z=0}$ is the wall heat flux.

The dimensionless form of the above equations can be written as

13 $$\:{Re}_{x}^{1/2}{C}_{fx}={A}_{1}\left(1+\frac{1}{\beta\:}\right){f}^{{\prime\:}{\prime\:}}\left(0\right),\:{Re}_{x}^{1/2}{C}_{fy}={A}_{1}\left(1+\frac{1}{\beta\:}\right){g}^{{\prime\:}{\prime\:}}\left(0\right),\:{Re}_{x}^{-1/2}{Nu}_{x}=-\left[A_4+\frac{4}{3Nr}(1+\left({\theta\:}_{w}-1\right)\theta\:(0){)}^{3}\right]{\theta\:}^{{\prime\:}}\left(0\right),$$

where the local Reynolds number $\:{Re}_{x}={U}_{w}\left(x\right)x/\nu_{f}\:$.

The dispersion of nanoparticles in the fluid changes the thermophysical properties of the base fluid. The relations between the thermophysical properties of the base fluid and nanoparticles are derived and available in the literature^48^. These relationships are given in Table 1.

**Table 1:** Relations between thermo-physical properties of base fluid and nano-particles.

Properties	Ternary nanofluid
Density	$\:{\rho\:}_{tnf}=(1-{\phi\:}_{1})\left\{\right(1-{\phi\:}_{2}\left)\right[(1-{\phi\:}_{3}){\rho\:}_{f}+{\phi\:}_{3}{\rho\:}_{3}]+{\phi\:}_{2}{\rho\:}_{2}\}+{\phi\:}_{1}{\rho\:}_{1}$
Heat capacity	$\:{\left(\rho\:{c}_{p}\right)}_{tnf}=(1-{\phi\:}_{1})\left\{\right(1-{\phi\:}_{2}\left)\right[(1-{\phi\:}_{3}){\left({\rho\:c}_{p}\right)}_{f}+{\phi\:}_{3}{\left(\rho\:{c}_{p}\right)}_{3}]+{\phi\:}_{2}{\left(\rho\:{c}_{p}\right)}_{2}\}+{\phi\:}_{1}{\left(\rho\:{c}_{p}\right)}_{1}$
Viscosity	$\:{\mu\:}_{tnf}=\frac{\mu\:_f}{(1-{\phi\:}_{1}{)}^{2.5}(1-{\phi\:}_{2}{)}^{2.5}(1-{\phi\:}_{3}{)}^{2.5}}$
Thermal conductivity	$\:{K}_{tnf}=\left[\frac{{K}_{1}+2{K}_{hnf}-2{\phi\:}_{1}\left({K}_{hnf}-{K}_{1}\right)}{{K}_{1}+2{K}_{hnf}+{\phi\:}_{1}\left({K}_{hnf}-{K}_{1}\right)}\right]\left[\frac{{K}_{2}+2{K}_{nf}-2{\phi\:}_{2}({K}_{nf}-{K}_{2})}{{K}_{2}+2{K}_{nf}+{\phi\:}_{2}({K}_{nf}-{K}_{2})}\right]\left[\frac{{K}_{3}+2{K}_{f}-2{\phi\:}_{3}({K}_{f}-{K}_{3})}{{K}_{3}+2{K}_{f}+{\phi\:}_{3}({K}_{f}-{K}_{3})}\right]$
Electrical conductivity	$\:{\sigma\:}_{tnf}=\left[\frac{{\sigma\:}_{1}+2{\sigma\:}_{hnf}-2{\phi\:}_{1}({\sigma\:}_{hnf}-{\sigma\:}_{1})}{{\sigma\:}_{1}+2{\sigma\:}_{hnf}+{\phi\:}_{1}({\sigma\:}_{hnf}-{\sigma\:}_{1})}\right]\left[\frac{{\sigma\:}_{2}+2{\sigma\:}_{nf}-2{\phi\:}_{2}({\sigma\:}_{nf}-{\sigma\:}_{2})}{{\sigma\:}_{2}+2{\sigma\:}_{nf}+{\phi\:}_{2}({\sigma\:}_{nf}-{\sigma\:}_{2})}\right]\left[\frac{{\sigma\:}_{3}+2{\sigma\:}_{f}-2{\phi\:}_{3}({\sigma\:}_{f}-{\sigma\:}_{3})}{\sigma\:3+2{\sigma\:}_{f}+{\phi\:}_{3}({\sigma\:}_{f}-{\sigma\:}_{3})}\right]$

The numerical values of kerosene oil and nano-particles $\:(F{e}_{3}{O}_{4},$ *Co,* and * Ni)* used in simulations are tabulated in Table 2.

**Table 2:** Thermophysical properties^48–50^.

Physical Properties	Kerosene	$\:\boldsymbol{F}{\boldsymbol{e}}_{3}{\boldsymbol{O}}_{4}$	Cobalt	Nickel
$\:{C}_{P\:}[J/kgK]$	* 2090*	* 670*	* 420*	* 445*
$\:\rho\:[kg/{m}^{3}]$	* 780*	* 5180*	8900	* 8089*
* K[W/mK]*	* 0.149*	* 80*	100	* 91*
$\:\sigma\:[W/mK]$	$\:6\:\mathrm{x}\:{10}^{-10}$	$\:0.112\:\mathrm{x}\:{10}^{6}$	$\:1.602\:\mathrm{x}\:{10}^{7}$	$\:1.43\:\mathrm{x}\:{10}^{7}$
$\:\mathrm{P}\mathrm{r}$	* 23.0114*	* -*	* -*	* -*

## Numerical scheme and method of solution

The numerical method used to find the numerical solutions of the problems under consideration is BVP4C. For details, see references^51–55^. To proceed with numerical solutions, let us assume

$$\:f={f}_{1\:},{f}^{{\prime\:}}={f}_{\begin{array}{c}2\\\:\:\end{array}},{f}^{{\prime\:}{\prime\:}}={f}_{3},g={f}_{4},{g}^{{\prime\:}}={f}_{5},{g}^{{\prime\:}{\prime\:}}={f}_{6}$$

15 $$\:\theta\:={f}_{7},{\theta\:}^{{\prime\:}}={f}_{8}$$

By using Eq. (15), the following set of equations is obtained with the transformed boundary conditions:

16 $$\:{f_1}^{{\prime\:}}={f}_{\begin{array}{c}2\\\:\:\end{array}},{f_2}^{{\prime\:}}={f}_{3},$$

17 $$\:{{f}^{{\prime\:}}}_{3}=-\left(\frac{{A}_{2}}{{A}_{1}}\right)\left(\frac{\beta\:}{\beta\:+1}\right)\left(\left(f_1+f_4\right){f_3}-\left(1+Fr\right){{f_2}}^{2}-\left({\left(\frac{{A}_{3}}{{A}_{2}}\right)M}^{2}+\frac{{A}_{1}}{{A}_{2}}\lambda\:\right){f_2}\right),$$

18 $$\:{f_4}^{{\prime\:}}={f}_{5},\:{f_5}^{{\prime\:}}={f}_{6},$$

19 $$\:{f^{\prime\:}}_{6}=-\left(\frac{{A}_{2}}{{A}_{1}}\right)\left(\frac{\beta\:}{\beta\:+1}\right)\left(\left(f_1+f_4\right){f_6}-\left(1+Fr\right){{f_5}}^{2}-\left(\left(\frac{{A}_{3}}{{A}_{2}}\right){M}^{2}+\frac{{A}_{1}}{{A}_{2}}\lambda\:\right){f_5}\right),$$

20 $$\:{f_7}^{{\prime\:}}={f}_{8},$$

$$\:{f^{\prime\:}}_{8}=-{\left[\left({{A}_{4}}+\left(\frac{4}{3{Nr}}\right)\left(1+\left({\theta\:}_{w}-1\right){f_7}\right)^{3}\right)\right]}^{-1}\Big[A_5\mathrm{Pr}\left(f_1+f_4\right)f_8+\mathrm{P}\mathrm{r}Bf_7\:$$

21 $$\:+\mathrm{P}\mathrm{r}{M}^{2}\left({Ec}_{x}{{f_2}}^{2}+{Ec}_{y}{{f_5}}^{2}\right)+\frac{4}{3Nr}(1+\left({\theta\:}_{w}-1\right)f_7\:{)}^{2}\left({\theta\:}_{w}-1\right){f_8}^{2}\Big],$$

22 $$\:{f}_{a}\left(1\right)-0,{f}_{a}\left(2\right)-1,{f}_{b}\left(2\right)-0,\:{f}_{a}\left(4\right)-0,{f}_{a}\left(5\right)-\alpha\:,\:{f}_{b}\left(5\right)-0,{f}_{a}\left(7\right)-1,{f}_{b}\left(7\right)-0.$$

### Error analysis at different mesh sizes

**Fig. 2:**
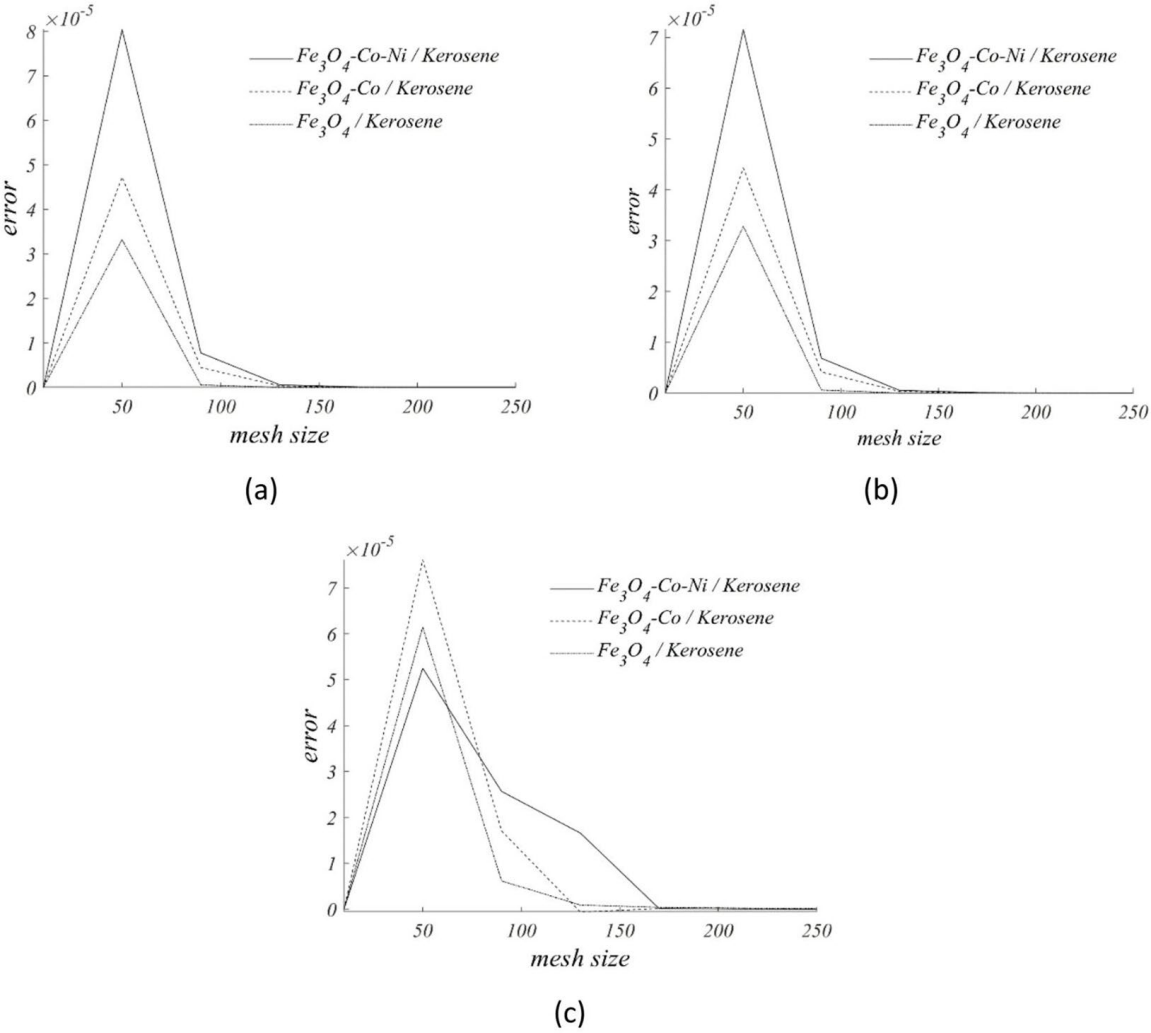
**(a**,** b**,** c)** An error analysis for $\:{Re}_{x}^{1/2}{C}_{fx}$, $\:{Re}_{x}^{1/2}{C}_{fy}$ and $\:{Re}_{x}^{-1/2}{Nu}_{x}$ for different grid sizes.

The numerical methods are reliable and authentic only when error approaches to zero. The error in the present case is defined by

23 $$\:Error=\left|{U}_{h}-{U}_{k}\right|$$

where values $\:{U}_{h}$ and $\:{U}_{k}$ are computed at mesh sizes * h* and * k* respectively. Here in case, skin friction coefficients in * x*- and * y*-directions and the local Nusselt number are computed at different number of elements (different grid sizes). The error defined in Eq. (23) is computed for skin friction coefficient and the local Nusselt number. It is obvious in Figs. 2(a, b, c) shows that error approaches zero as grid sizes decreases. These Figures show that error related skin friction coefficient (Figs. 2(a, b)) becomes almost zero at 150 elements and error related to the local Nusselt number (Fig. 2c) becomes zero when domain is discretized into 200 elements. The Figures also shows that solution for the skin friction coefficients and the local Nusselt number at 150and 200 elements respectively. Hence, all post processing computations are carried out at 200 elements.

### Validation of results

It is worth mentioning that the present modelled problem (8)-(11) is reducible to the already published study by Shehzad et al.^25^, Ganesh et al.^56^ and Muhammad et al.^57^ when $\:\beta\:\to\:\infty\:$, * M=0*, $\:\lambda\:=0$, * Fr=0* and $\:{\phi\:}_{1}={\phi\:}_{2}={\phi\:}_{3}=0.$ The present results are validated by giving a comparison between the present results (as reduced case) and the results of Shehzad et al.^25^, Ganesh et al.^56^ and Muhammad et al.^57^. This validation is given in Table 3.

**Table 3:** Numerical values of $\:{f}^{{\prime\:}{\prime\:}}\left(0\right)\:$ and $\:\:{g}^{{\prime\:}{\prime\:}}\left(0\right)$ for different values of $\:\alpha\:$ when $\:\beta\:\to\:\infty\:$, * M=0*, $\:\lambda\:=0$, $\:Fr=0\:$and $\:{\phi\:}_{1}={\phi\:}_{2}={\phi\:}_{3}=0$.

$\:\boldsymbol{\alpha\:}$	Shehzad et al.^25^		Ganesh et al.^56^		Muhammad et al.^57^		Present results	
	$\:-f^{{\prime\:}{\prime\:}}\left(0\right)$	$\:-g^{{\prime\:}{\prime\:}}\left(0\right)$	$\:-f^{{\prime\:}{\prime\:}}\left(0\right)$	$\:-g^{{\prime\:}{\prime\:}}\left(0\right)$	$\:-f^{{\prime\:}{\prime\:}}\left(0\right)$	$\:-g^{{\prime\:}{\prime\:}}\left(0\right)$	$\:-f^{{\prime\:}{\prime\:}}\left(0\right)$	$\:-g^{{\prime\:}{\prime\:}}\left(0\right)$
0.0	1	0	1	0	1	0	1.0004836283	0.0000000149
0.25	1.04881	0.19457	1.04881	0.19457	1.04881	0.19457	1.0490595744	0.1946994164
0.50	1.09309	0.46522	1.09309	0.46522	1.09308	0.46523	1.0932409046	0.4653157296
0.75	1.13450	0.79462	1.13450	0.79462	1.13452	0.79462	1.1345740985	0.7946955184
1.0	1.17372	1.17372	1.17372	1.17372	1.17372	1.17372	1.1737794212	1.1737794212

## Detailed discussion on data related to visualization

Three-dimensional transport equations dealing with the influence of multi-nanoscale particles on thermal enhancement in Casson fluid exhibiting yield stress in the presence of a magnetic field, Joule heating, and non-linear Darcy-Forchheimer porous medium are numerically solved, and computed solutions are further used for studying the quantities of engineering interest. A comprehensive comparative analysis on the improvements in the thermal performance of Casson fluid under the dispersion of three combinations of nanoparticles is presented. These combinations are: (i) $\:F{e}_{3}{O}_{4}$-Casson nanofluid, (ii) $\:F{e}_{3}{O}_{4}$-* Co*-Casson nanofluid, and (iii) $\:F{e}_{3}{O}_{4}$-* Co*-* Ni*-Casson nanofluid. This study aims to reveal which combination of nanoparticles plays a role in optimising the thermal performance of fluids. The detailed discussion based on animations is given below:

### Comparison among wall heat fluxes for $\:\boldsymbol{F}{\boldsymbol{e}}_{3}{\boldsymbol{O}}_{4}$-Casson nanofluid, $\:\boldsymbol{F}{\boldsymbol{e}}_{3}{\boldsymbol{O}}_{4}$-$\:\boldsymbol{C}\boldsymbol{o}$-Casson nanofluid and $\:\boldsymbol{F}{\boldsymbol{e}}_{3}{\boldsymbol{O}}_{4}$-$\:\boldsymbol{C}\boldsymbol{o}$-$\:\boldsymbol{N}\boldsymbol{i}$-Casson nanofluid

**Fig. 3:**
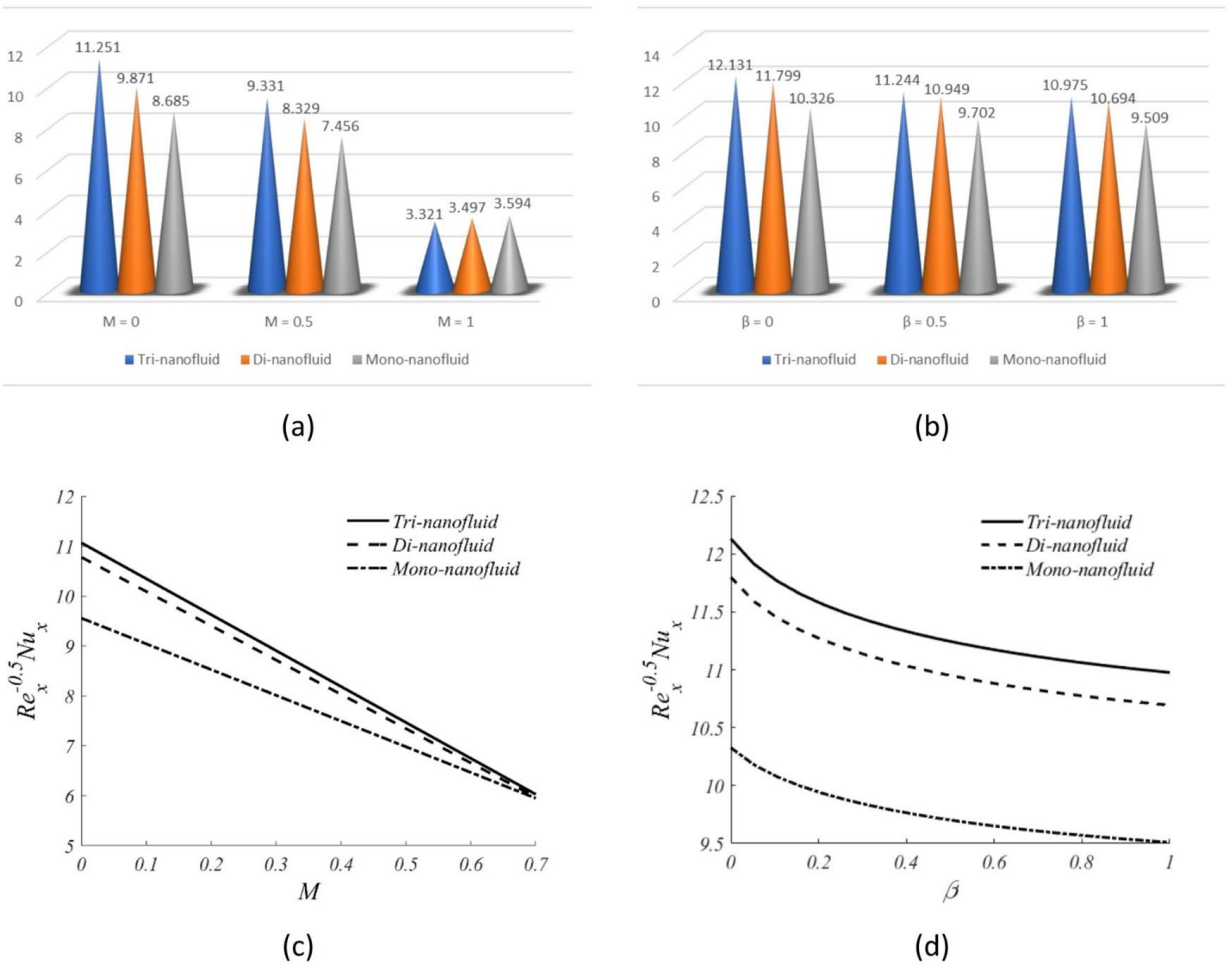
**(a**,** b**,** c**,** d)** Variation of $\:{R{e}_{x}}^{-1/2}N{u}_{x}\:$ against different values of Hartman number $\:M\:$ and Casson fluid parameter $\:\beta\:$ when * B=0.1,* *Nr=0.5,* $\:{\theta\:}_{w}=1.1,$ $\:{Ec}_{x}=0.1$ and $\:{Ec}_{y}=0.1$.

Three types of fluids, mono-nanofluid ($\:F{e}_{3}{O}_{4}$-kerosene oil), di-nanofluid ($\:F{e}_{3}{O}_{4}$-* Co*-kerosene oil), and tri-nanofluid ($\:F{e}_{3}{O}_{4}$-* Co*-* Ni***-**kerosene oil) are considered and the local Nusselt number (wall heat transport rate) for these considered fluids is calculated to compare their thermal performances. In addition to this, the behaviour of local Nusselt number for change in * M,* $\:\beta\:,$ *Nr*, $\:{\theta\:}_{w}$, * B*, $\:{\left(Ec\right)}_{x}$, and $\:({Ec)}_{y}\:$on the local Nusselt number is studied quantitatively qualitatively. For presenting data clearly and effectively in a simple manner, the bar graphs (Figs. 3(a, b, c, d) to Fig. 5(a, b), and Fig. 6) have their significance. The numerical computations are listed in Table 4. It is evident from Table 4 that an increase in the intensity of magnetic induction is responsible for a decrease in the wall heat transfer rate (the local Nusselt number). The reason behind this notable decrease in the local Nusselt number ( $\:{R{e}_{x}}^{-\frac{1}{2}}N{u}_{x}$ ) is that an increase in magnetic induction results in an increase in Joule heating (a process which dissipates heat due to conversion of electrical current into heat) due to which fluid’s temperature rises and its capacity to carry heat reduces. Therefore, it is concluded that the working fluid should not be heat dissipative or ta magnetic field should not be applied to the working fluid so that it cannot produce heat through the Ohmic process. The local Nusselt number decreases when the heat generation parameter is increased. It means that thermal performance is compromised due to the generation of heat in the fluid. Hence, it is concluded that fluid should not be heat generating. It is also evident that fluid should be the heat absorbing as in case of heat absorption, thermal performance increases. Thus, an optimized thermal performance, the characteristic of fluid to be heat absorbing is a favourable mechanism. An increase in $\:{\left(Ec\right)}_{x}$ and $\:{\left(Ec\right)}_{y}$ is also responsible of a decrease in heat transfer rate. Thus, for an increased thermal performance, fluid should be the least ohmic dissipative. Table 4 also depicts that kerosene oil with tri-nano particles ($\:F{e}_{3}{O}_{4}$-* Co*-* Ni***-**kerosene oil) gives the maximum thermal performance. Thus, $\:F{e}_{3}{O}_{4}$-* Co*-* Ni*-kerosene oil is the best selection for optimal heat transport in comparison with $\:F{e}_{3}{O}_{4}$-kerosene oil and $\:F{e}_{3}{O}_{4}$-* Co*-kerosene oil.

The graphs provided through Figs. 3(a, b, c, d) to Fig. 6 provide for the quick review of data size to compare the cases of the highest and the lowest rise in the thermal performances of sample fluids due to dispersion of different combinations of nanoparticles. The quantitative effectiveness is studied versus related parameters for side-by-side comparison using visualization through bar graphs of wall heat transfer rate. Numerical data in Table 4 and statistical representation in Figs. 3(a, b, c, d) to Figs. 6, clearly show that the tri-nanoparticles ($\:F{e}_{3},\:Co$, * Ni* ) are responsible for an optimized effectiveness in thermal enhancement in wall heat transfer rate in kerosine oil. It is also noted that for higher values of * M*, the thermal performance of the tri-nano-kerosine oil is compromised, which indicates that tri-nano-kerosine oil generates higher heat due to the ohmic dissipation process in comparison with mono and di-nano-kerosine oil. Thus, it is concluded that tri-nanoparticles in kerosine oil are effective only relative to mono-, and di-nanoparticles in kerosine oil if kerosine oil is not exposed to the magnetic field (see Fig. 3a). Therefore, in thermal and cooling systems, automotive mobiles, and other industrial processes (where thermal enhancement is required), kerosine oil with nanoparticles should not be exposed to the magnetic field. Figure 3b depicts that the yield stress is responsible for decreasing the wall transfer rate. Bar graphs in Fig. ﻿4b have given the prediction that heat generation reduces the thermal heat transfer rate as an increase, whereas the reverse behavior of the thermal radiation parameter in comparison to the behavior of the heat generation parameter is noticed (Fig. ﻿4a). Figure 3c and d, respectively, indicate the influence of Hartmann number and Casson fluid parameter on the local Nusselt number. It is visualized from Figs. 5(a, b) that the viscous dissipation phenomenon is not a supportive phenomenon concerning thermal enhancement. It is also found from the bar graph given in Fig. 6 that an enhancement in the temperature of the surface results in a rise in the wall heat flux.

**Fig. 4:**
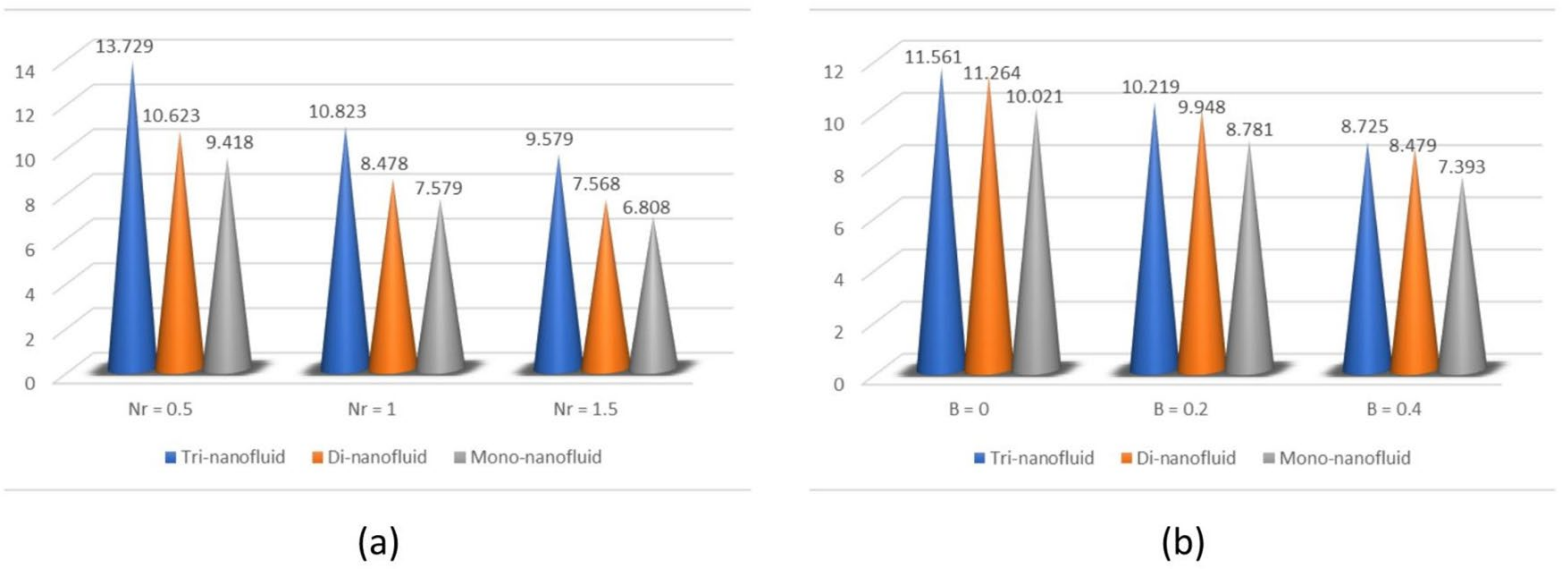
**(a**,** b)** Variation of $\:{R{e}_{x}}^{-1/2}N{u}_{x}\:$ against different values of radiation parameter $\:Nr\:$ and heat generation parameter * B* when * M=0.1,* $\:\beta\:=1.2,$ $\:{\theta\:}_{w}=1.1,$ $\:{Ec}_{x}=0.1$ and $\:{Ec}_{y}=0.1$.

**Fig. 5:**
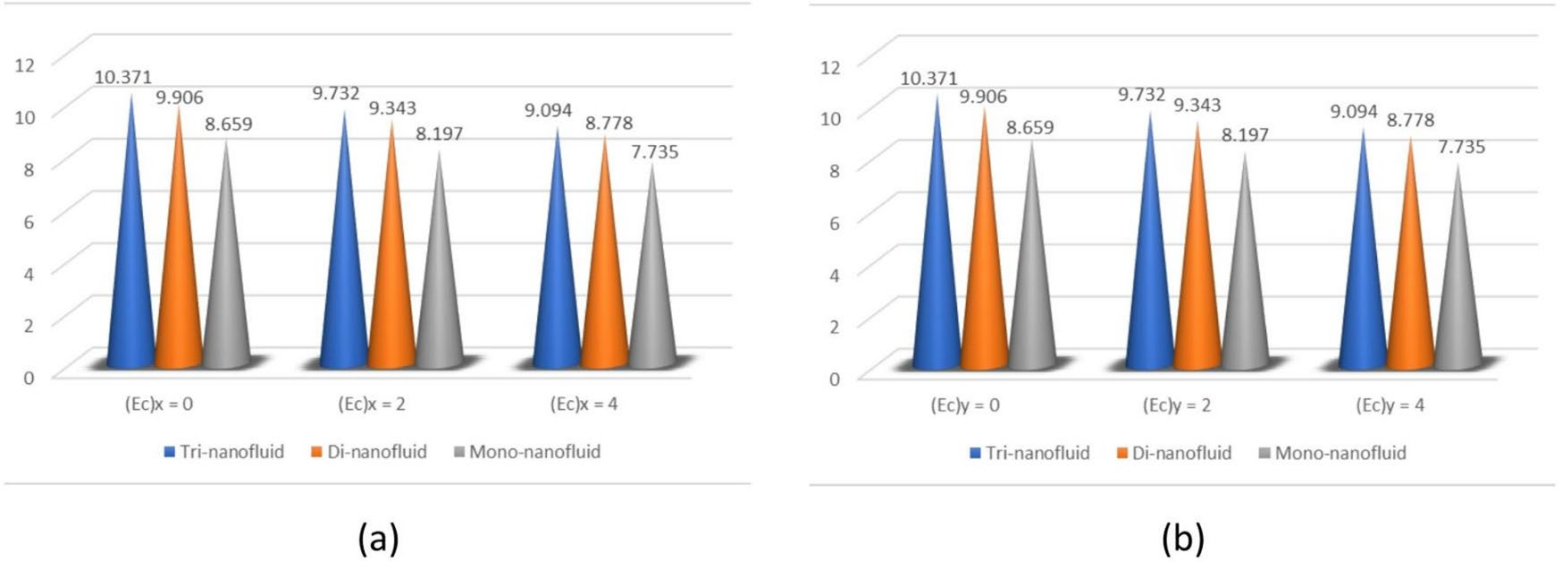
**(a**,** b)** Variation of $\:{R{e}_{x}}^{-1/2}N{u}_{x}\:$ against different values of Eckert numbers $\:{Ec}_{x}$ and $\:{Ec}_{y}$ when * M=0.1,* $\:\beta\:=1.2,$ $\:{\theta\:}_{w}=1.1,\:B=0.1,$ and * Nr=0.5*.

**Fig. 6:**
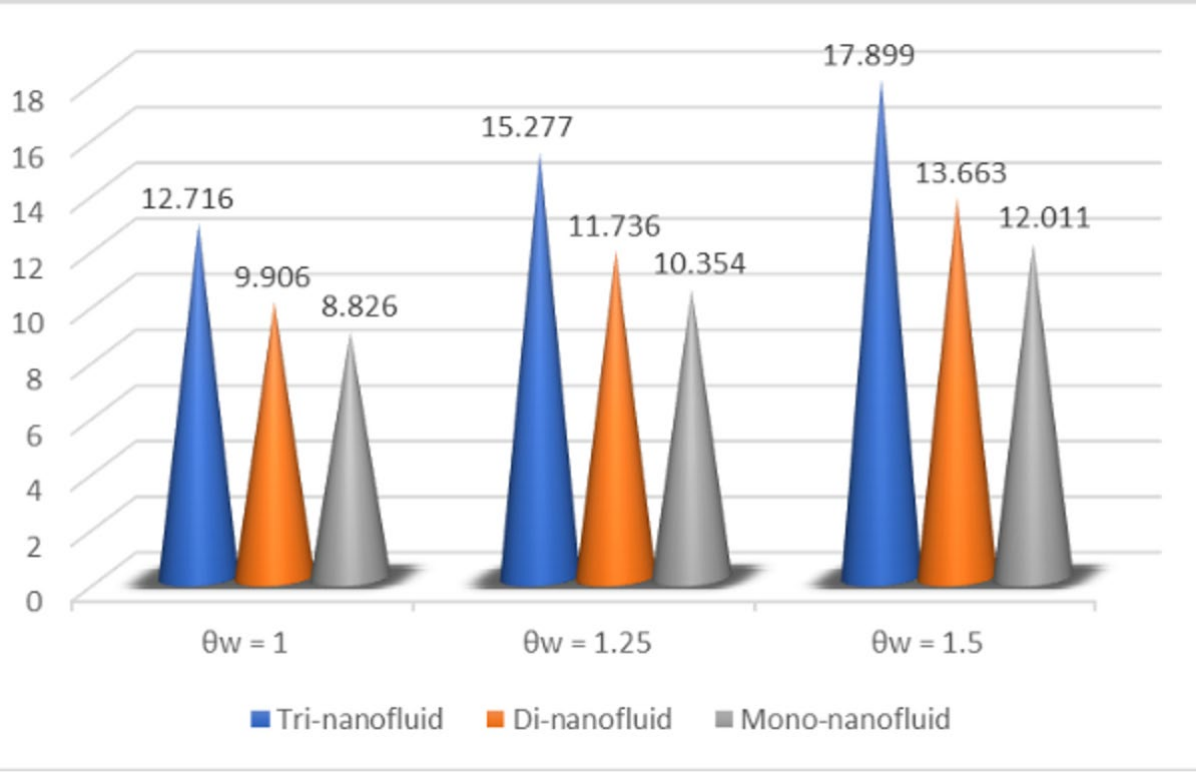
Variation of $\:{R{e}_{x}}^{-1/2}N{u}_{x}\:$ against different values of $\:{\theta\:}_{w}$ when * B=0.1,* *Nr=0.5,* *M=0,* $\:\beta\:=1.2,$ *,* $\:{Ec}_{x}=0.1$ and $\:{Ec}_{y}=0.1$.

**Table 4:** $\:{R{e}_{x}}^{-1/2}N{u}_{x}$ for different values of * M*, $\:\beta\:$, * Nr*, $\:{\theta\:}_{w}$, * B*, $\:{\left(Ec\right)}_{x}$ and $\:({Ec)}_{y}.$.

$\:\boldsymbol{M}$	$\:\boldsymbol{\beta\:}$	$\:\boldsymbol{N}\boldsymbol{r}$	$\:{\boldsymbol{\theta\:}}_{\boldsymbol{w}}$	$\:\boldsymbol{B}$	$\:{\left(\boldsymbol{E}\boldsymbol{c}\right)}_{\boldsymbol{x}}$	($\:{\boldsymbol{E}\boldsymbol{c})}_{\boldsymbol{y}}$	$\:\boldsymbol{F}{\boldsymbol{e}}_{3}{\boldsymbol{O}}_{4}-\boldsymbol{C}\boldsymbol{o}-\boldsymbol{N}\boldsymbol{i}-\mathbf{k}\mathbf{e}\mathbf{r}\mathbf{o}\mathbf{s}\mathbf{e}\mathbf{n}\mathbf{e}\:\mathbf{o}\mathbf{i}\mathbf{l}$	$\:\boldsymbol{F}{\boldsymbol{e}}_{3}{\boldsymbol{O}}_{4}-\boldsymbol{C}\boldsymbol{o}-$ kerosene oil	$\:\boldsymbol{F}{\boldsymbol{e}}_{3}{\boldsymbol{O}}_{4}$- kerosene oil
0	1.2	0.5	1.1	0.1	0.1	0.1	10.9680381822	9.6165928414	8.4519716190
0.5							8.9851120075	8.0213966038	7.1768716299
1							2.8013620556	3.0413619380	3.1890682543
0.1	0	0.5	1.1	0.1	0.1	0.1	12.1304863128	11.7990749085	10.3264283975
	0.5						10.9699625528	10.6816806168	9.4506292146
	1.0						10.6348406290	10.3594025360	9.1949732936
0.1	1.2	0.5	1.1	0.1	0.1	0.1	13.3763795797	10.3448854015	9.1625041919
		1					10.6019459288	8.3046681794	7.4195498383
		1.5					9.4020751681	7.4294484104	6.6814633406
0.1	1.2	0.5	1	0.1	0.1	0.1	12.4067817007	9.6611170782	8.5995317595
			1.25				14.8515115041	11.4018991672	10.0483913388
			1.5				17.3261608870	13.2123082855	11.5981657242
0.1	1.2	0.5	1.1	-0.4	0.1	0.1	13.7286115697	13.3931248771	12.0235758950
				-0.2			12.5675380132	12.2539554517	10.9569947214
				0			11.3028409865	11.0126072770	9.7912890667
				0.2			9.9046991872	9.6395202935	8.4967416546
				0.4			8.3274708718	8.0893578870	7.0271332155
0.1	1.2	0.5	1.1	0.1	0	0.1	10.1057112551	9.6463710353	8.4256347900
					2		9.4409905353	9.0582097171	7.9427511797
					4		8.7753636352	8.4692754192	7.4592531405
0.1	1.2	0.5	1.1	0.1	0.1	0	10.1057112551	9.6463710353	8.4256347900
						2	9.4409905353	9.0582097171	7.9427511797
						4	8.7753636352	8.4692754192	7.4592531405

### Study of wall shear stresses for $\:\boldsymbol{F}{\boldsymbol{e}}_{3}{\boldsymbol{O}}_{4}$-Casson nanofluid, $\:\boldsymbol{F}{\boldsymbol{e}}_{3}{\boldsymbol{O}}_{4}$-$\:\boldsymbol{C}\boldsymbol{o}$-Casson nanofluid and$\:\:\boldsymbol{F}{\boldsymbol{e}}_{3}{\boldsymbol{O}}_{4}$-$\:\boldsymbol{C}\boldsymbol{o}$-$\:\boldsymbol{N}\boldsymbol{i}$-Casson nanofluid

In this study, two tangential stresses (in * x-* and * y-*directions) are derived or computed, and normalized. These normalized tangential stress against related parameters (* M,* $\:\beta\:,$ $\:\lambda\:$ and * Fr*). The behaviour of these shear stresses (tangential stress) is computed, and numerical values are displayed in Tables 5 and 6. Table 5 is about tangential stress in the * x-*direction, whereas Table 6 is about the behaviour of tangential stress in * y-*direction. It is evident from Tables 5 and 6 that wall shear stresses increase because of the presence of a porous medium. It is also noted that an increase in the strength of magnetic results in an increase in wall shear stress (tangential stress at the solid surface). A Porous medium exerts a resistive force on to flow of the fluid. This resistive force is proportional to the velocity of the fluid (see the second last and last term in Eq. $\:\left(3\right)$). Hence, the higher the velocity of the fluid higher will be this resistive force. Since tri-nanofluid has the highest velocity induced by the motion of the solid boundary. Therefore, the highest drag force is the reason tri-nanofluid flow experiences is the highest opposing force as shown in tigure 6b. Further, Darcy porous medium term involved viscosity which is directly related to retardation (opposing force) exerted by the Darcy porous medium (see second last term in Eq. $\:\left(3\right)$). Since the viscosity of the nanofluid is modified in terms of volume fractions of nanoparticles (see Table 1). Therefore, effective viscosity increases due to the dispersion of nanoparticles. Hence, resistance to fluid by porous medium is the highest for the case of tri-nanoparticles. This is why tri-nanofluid flow experiences the highest opposing effects. Further, an increase in the yield stress, wall shear stress. It is also evident from Tables 5 and 6 that tri-nanofluid ($\:F{e}_{3}{O}_{4}$-* Co*-* Ni*-kerosene oil) exerts the highest wall stress in comparison with $\:F{e}_{3}{O}_{4}$-kerosene oil and $\:F{e}_{3}{O}_{4}$-* Co*-kerosene oil.

**Fig. 7:**
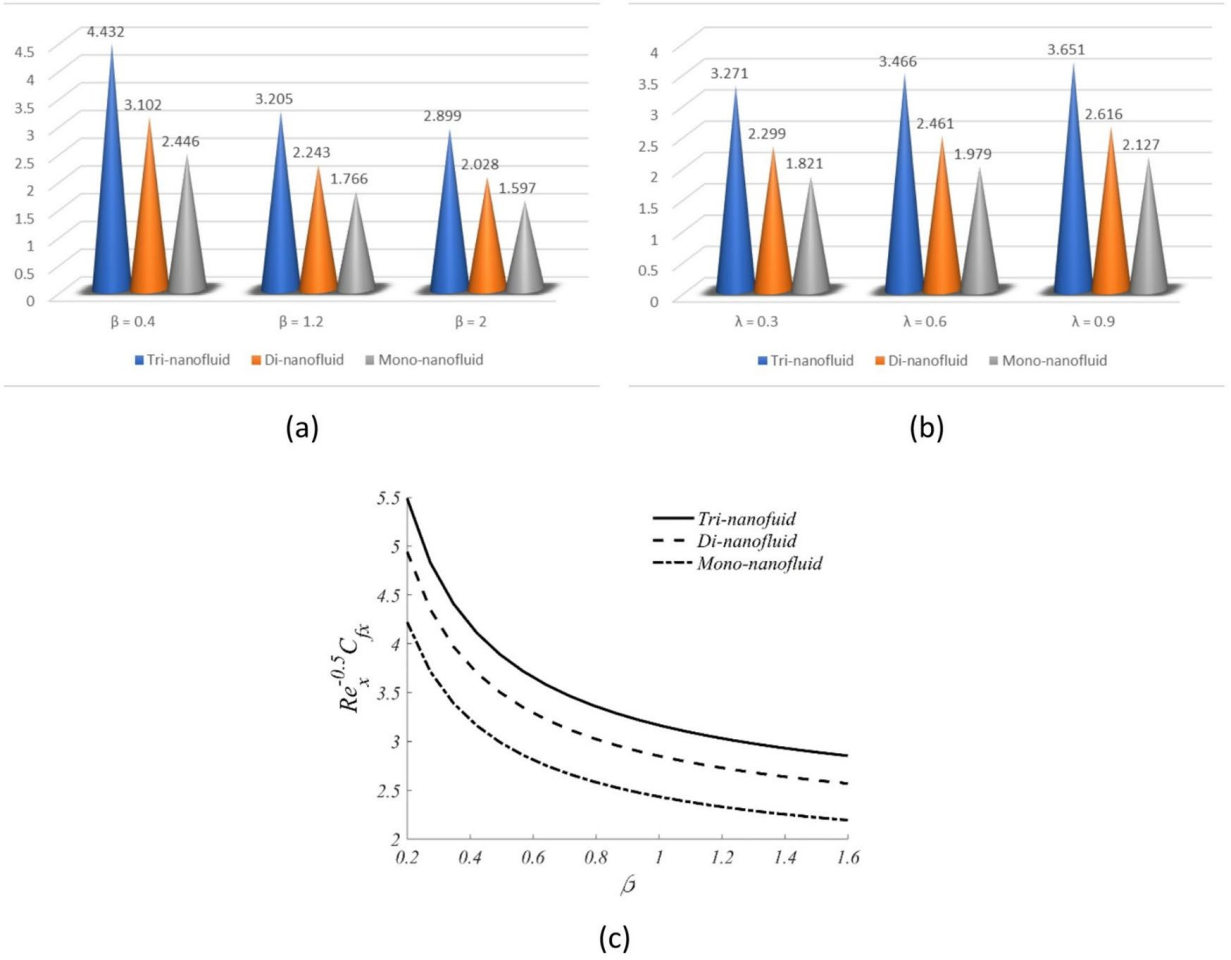
**(a**,** b**,** c)** Variation of $\:{-R{e}_{x}}^{1/2}{C}_{fx}\:\:$ against different values of Casson fluid parameter $\:\beta\:$ and Darcy porous medium parameter $\:\lambda\:$ when * M=0.1,* $\:\alpha\:=0.5,$ *B=0.1,* *Fr=0.1,* $\:{\theta\:}_{w}=1.1,Sc=5$, $\:\stackrel{-}{\sigma\:}=0.1\:$, $\:E=1.0\:,$ and $\:\delta\:=\:$0.2.

**Fig. 8:**
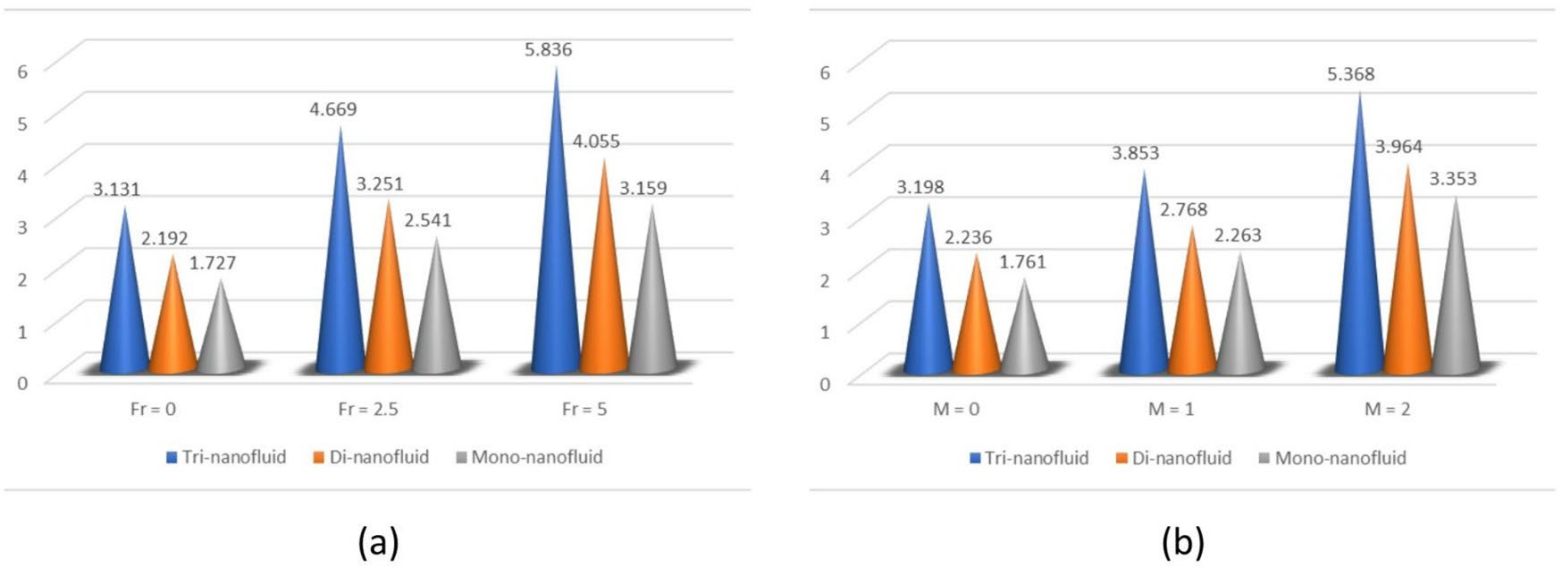
**(a**,** b)** Variation of $\:{-R{e}_{x}}^{1/2}{C}_{fx}\:\:$ against different values of Forchheimer * Fr* and Hartman number * M* when $\:\beta\:=1.2,$ $\:\alpha\:=0.5,$ *B=0.1,* $\:\lambda\:=0.2,$ $\:{\theta\:}_{w}=1.1,Sc=5$, $\:\stackrel{-}{\sigma\:}=0.1\:$, $\:E=1.0\:,$ and $\:\delta\:=\:$0.2.

**Fig. 9:**
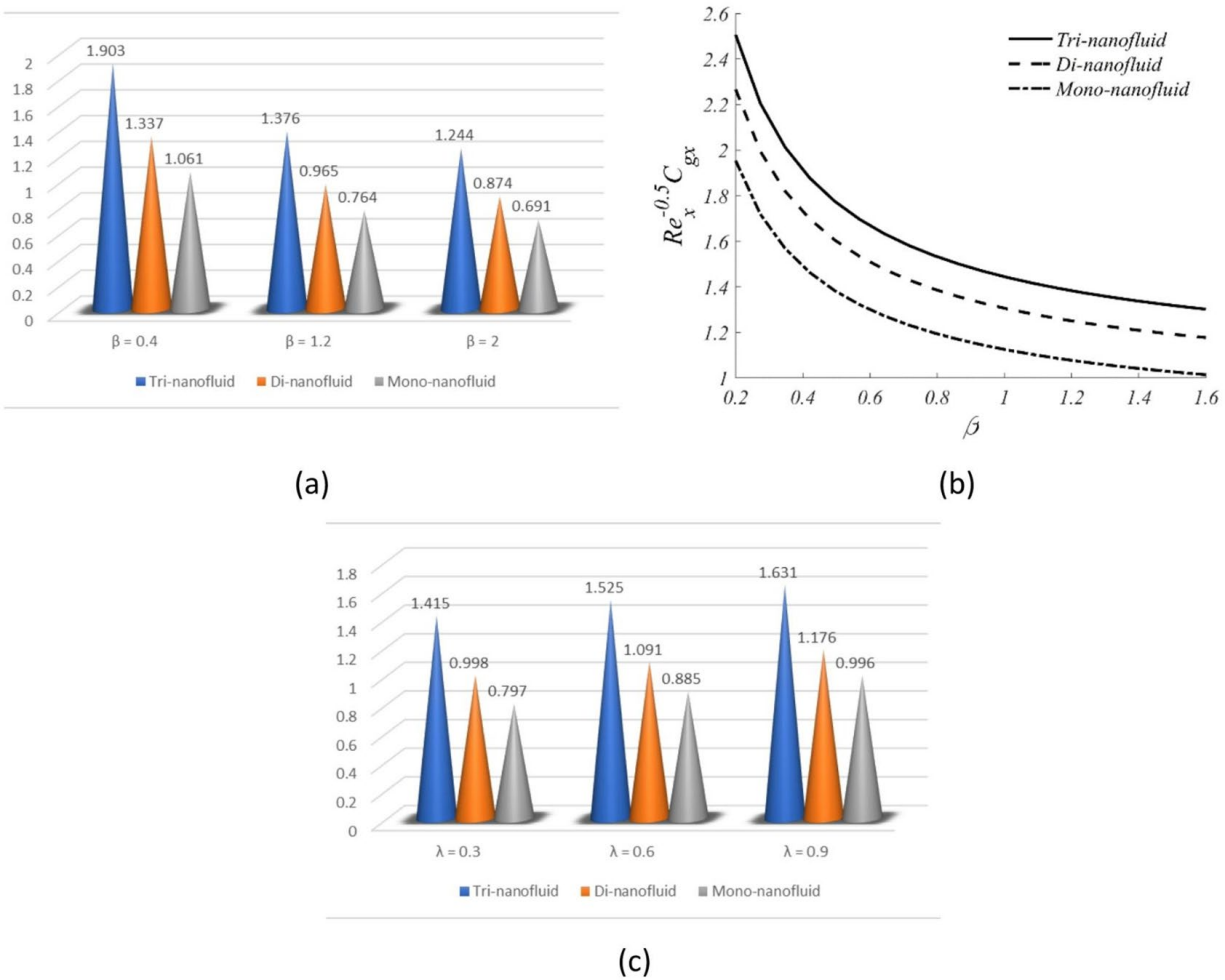
**(a**,** b**,** c)** Variation $\:{-R{e}_{x}}^{1/2}{C}_{fy}\:$ against different values of Casson fluid parameter $\:\beta\:$ and Darcy porous medium parameter $\:\lambda\:$ when * M=0.1,* $\:\alpha\:=0.5,$ *B=0.1,* *Fr=0.1,* $\:{\theta\:}_{w}=1.1,Sc=5$, $\:\stackrel{-}{\sigma\:}=0.1\:$, $\:E=1.0\:,$ and $\:\delta\:=\:$0.2.

**Fig. 10:**
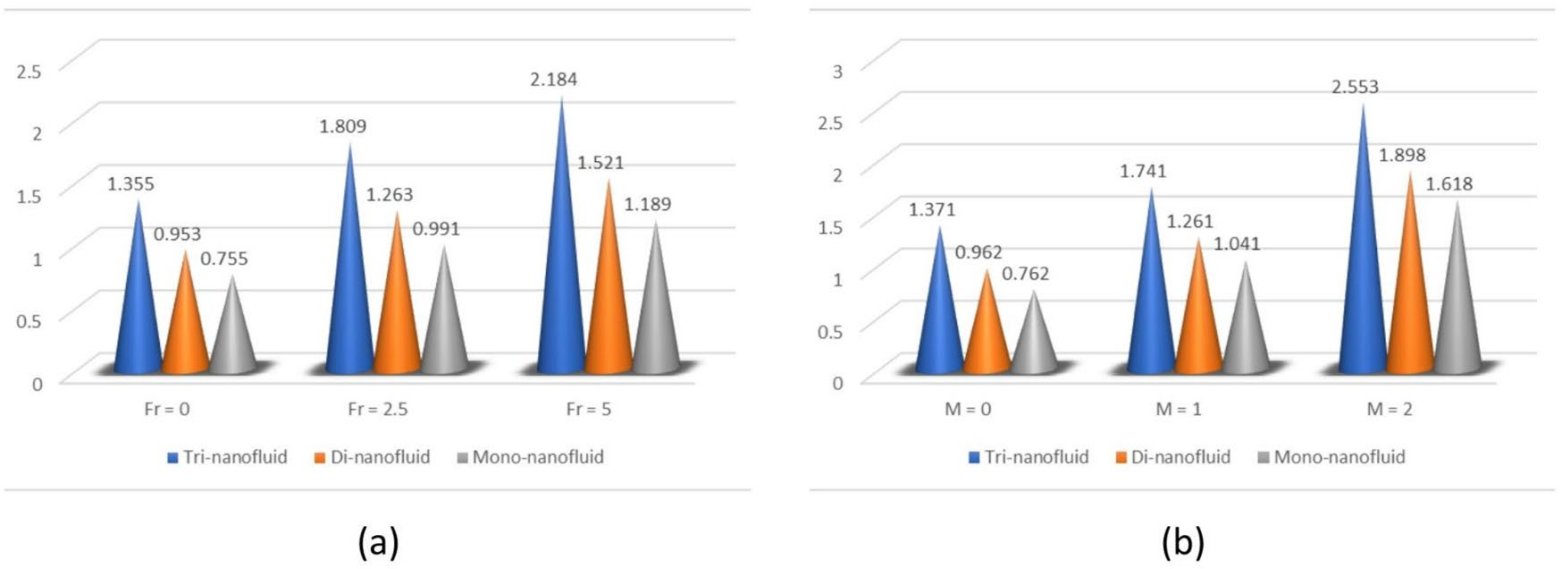
**(a**,** b)** Variation $\:{-R{e}_{x}}^{1/2}{C}_{fy}\:$ against different values of Forchheimer parameter * Fr* and Hartman number * M* when $\:\beta\:=1.2,$ $\:\lambda\:=0.2,$ $\:\alpha\:=0.5,$ *B=0.1,* $\:{\theta\:}_{w}=1.1,$, * Sc=5*, $\:\stackrel{-}{\sigma\:}=0.1\:$, $\:E=1.0\:,$ and $\:\delta\:=\:$0.2.

As it is pointed out, bar graphs help in quickly reviewing the data size and provide a quick comparison. Therefore, Figs. 7(a, b, c) to Fig. 10(a, b) are the graphs (for skin friction coefficients in tangential directions) for trend identification versus related parameters, as bar graphs make it possible to compare categories precisely by giving the heights and lengths of the bar graphs numerical values. When precise figures are necessary in addition to visual comparisons, this is helpful. The bar graphs in Figs. 7(a, b, c) and 9(a, b) indicate that the wall shear stresses decrease against an increase in yield stress and Darcy porous medium parameters. However, graphs in Figs. 8(a, b, c), and 10(a, b) give the predictions about the rising effects of the magnetic field and the Forchheimer porous medium on wall shear stress in * x-* and * y-*directions.

**Table 5:** $\:{-R{e}_{x}}^{1/2}{C}_{fx}\:$ for different values of * M*, $\:\beta\:$, $\:\lambda\:$ and * Fr.*.

$\:\boldsymbol{M}$	$\:\boldsymbol{\beta\:}$	$\:\boldsymbol{\lambda\:}$	$\:\boldsymbol{F}\boldsymbol{r}$	$\:\boldsymbol{F}{\boldsymbol{e}}_{3}{\boldsymbol{O}}_{4}-\boldsymbol{C}\boldsymbol{o}-\boldsymbol{N}\boldsymbol{i}-\mathbf{k}\mathbf{e}\mathbf{r}\mathbf{o}\mathbf{s}\mathbf{e}\mathbf{n}\mathbf{e}\:\mathbf{o}\mathbf{i}\mathbf{l}$	$\:\boldsymbol{F}{\boldsymbol{e}}_{3}{\boldsymbol{O}}_{4}-\boldsymbol{C}\boldsymbol{o}-$ kerosene oil	$\:\boldsymbol{F}{\boldsymbol{e}}_{3}{\boldsymbol{O}}_{4}$- kerosene oil
0	1.2	1.2	0.1	3.8217234499	2.7554673367	2.2594636994
1				4.3875961184	3.2051318162	2.6727863309
2				5.7652341309	4.2823493642	3.6434429804
0.4	0.4	1.2	0.1	5.4135020798	3.9137003002	3.2206735409
	1.2			3.9175907781	2.8320933481	2.3304288927
	2			3.5435839138	2.5617130017	2.1079367579
0.4	1.2	0.3	0.1	3.3755896929	2.3840653331	1.9026588228
		0.6		3.5649355695	2.5417703530	2.0547360773
		0.9		3.7452355043	2.6907012990	2.1967729098
0.4	1.2	1.2	0	4.2888843370	3.1424692210	2.6281672605
			2.5	5.5288685596	3.9655809928	3.2300529174
			5	6.5456143978	4.6495701903	3.7389060882

**Table 6:** $\:{-R{e}_{x}}^{1/2}{C}_{fy}\:$ for different values of * M*, $\:\beta\:$, $\:\lambda\:$ and * Fr.*.

* M*	$\:\beta\:$	$\:\lambda\:$	* Fr*	$\:F{e}_{3}{O}_{4}-Co-Ni-\mathrm{k}\mathrm{e}\mathrm{r}\mathrm{o}\mathrm{s}\mathrm{e}\mathrm{n}\mathrm{e}\:\mathrm{o}\mathrm{i}\mathrm{l}$	$\:F{e}_{3}{O}_{4}-Co-$ kerosene oil	$\:F{e}_{3}{O}_{4}$- kerosene oil
0	1.2	1.2	0.1	1.7239891225	1.2538245993	1.0392608799
1				2.0328129681	1.4972129926	1.2607398383
2				2.7619069899	2.0634186685	1.7669251648
0.4	0.4	1.2	0.1	2.4555096683	1.7907365522	1.4895035740
	1.2			1.7768945720	1.2957603839	1.0776970085
	2			1.6072548324	1.1720517477	0.9748039266
0.4	1.2	0.3	0.1	1.4735731984	1.0468417642	0.8422730866
		0.6		1.5807933064	1.1355797432	0.9271064996
		0.9		1.6815738606	1.2182003649	1.0051311775
0.4	1.2	1.2	0	1.9950740280	1.4737658528	1.2442801852
			2.5	2.3384218002	1.6996777398	1.4076826366
			5	2.6419386787	1.9013990820	1.5554325312

### Study of behaviour of velocity field versus key parameters

#### Influence of the change in magnetic induction on the flow of $\:\boldsymbol{F}{\boldsymbol{e}}_{3}{\boldsymbol{O}}_{4}$-Casson nanofluid, $\:\boldsymbol{F}{\boldsymbol{e}}_{3}{\boldsymbol{O}}_{4}$-$\:\boldsymbol{C}\boldsymbol{o}$-Casson nanofluid, and$\:\:\boldsymbol{F}{\boldsymbol{e}}_{3}{\boldsymbol{O}}_{4}$-$\:\boldsymbol{C}\boldsymbol{o}$-$\:\boldsymbol{N}\boldsymbol{i}$-Casson nanofluid

**Fig. 11:**
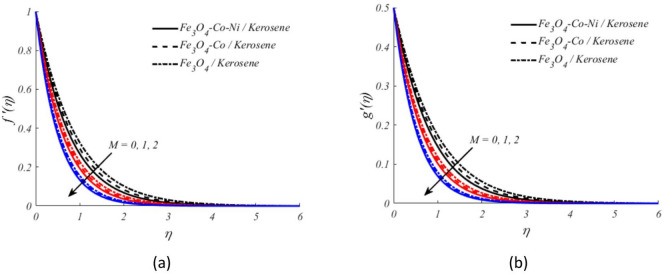
**(a**,** b)** Influence of Hartman number * M* on $\:{f}^{{\prime\:}}\left(\eta\:\right)$ and $\:{g}^{{\prime\:}}\left(\eta\:\right)$ when $\:\beta\:=1.2,$ $\:\lambda\:=1.2,$ $\:\alpha\:=0.5,$ *B=0.1,* *Fr=0.1,* *Nr=1.5,* $\:{\theta\:}_{w}=1.1,$ $\:{Ec}_{x}=0.5$ and $\:{Ec}_{y}=0.5$.

The magnetic induction parameter (Hartmann number) is varied, and animations are observed. The observed variations in velocity profiles under the influence of a magnetic field are reduced in Figs. 11(a, b). The behaviour of velocity profiles for the above-mentioned three types of fluids is studied, and a decrease in fluid motion due to an increase in a magnetic field is seen. From Figs. 11(a b) that the highest influence of the magnetic field on the flow of $\:F{e}_{3}{O}_{4}$-* Co*-* Ni*-Kerosene oil (Casson nanofluid) is noted. Thus, magnetic fields can be used to control the boundary layer flow. Figures 11(a, b) are tangential components of velocity, and both have a decreasing tendency versus an increase in the strength of the magnetic field.

#### Influence of yield stress on fluid motion

**Fig. 12:**
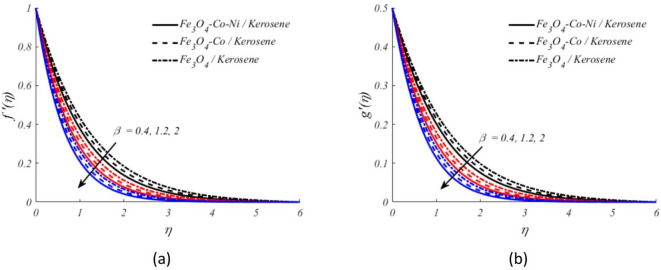
**(a**,** b)** Influence of Casson fluid parameter $\:\beta\:$ on $\:{f}^{{\prime\:}}\left(\eta\:\right)$ and $\:{g}^{{\prime\:}}\left(\eta\:\right)$ when * M=0.4,* $\:\lambda\:=1.2,$ $\:\alpha\:=0.5,$ *B=0.1,* *Fr=0.1,* *Nr=1.5,* $\:{\theta\:}_{w}=1.1,$ $\:{Ec}_{x}=0.5$ and $\:{Ec}_{y}=0.5$.

The parameter $\:\beta\:$ is the Casson fluid parameter, and it determines the impact of yield stress on fluid motion. The impact of $\:\beta\:$ on velocity components is animated in Figs. 12(a, b). These figures show a significant decrease in $\:f^{\prime\:}$ and $\:g^{\prime\:}$. Thus, fluids with higher yield stress move slower than fluids with lower yield. In other words, the yield stress can help in controlling the momentum boundary layer. Moreover, the fluid with three-type nanoparticles exerts the highest yield stress in comparison with di- and mono-nanoparticles.

#### Influence of porous medium on the fluid motion

**Fig. 13:**
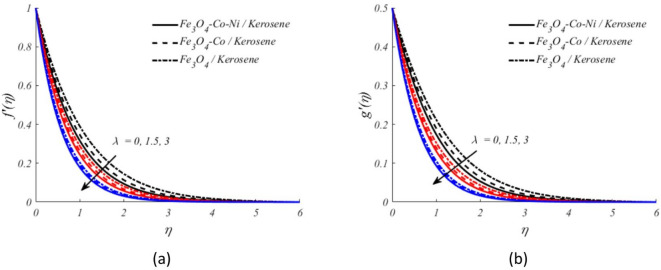
**(a**,** b)** Influence of Darcy porous medium parameter $\:\lambda\:$ on $\:{f}^{{\prime\:}}\left(\eta\:\right)$ and $\:{g}^{{\prime\:}}\left(\eta\:\right)$ when * M=0.4,* $\:\beta\:=1.2,$ $\:\alpha\:=0.5,$ *B=0.1,* *Fr=0.1,* *Nr=1.5,* $\:{\theta\:}_{w}=1.1,$ $\:{Ec}_{x}=0.5$ and $\:{Ec}_{y}=0.5$.

**Fig. 14:**
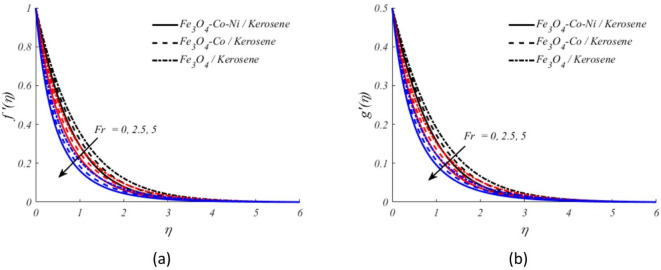
**(a**,** b)** Influence of Forchheimer parameter * Fr* on $\:{f}^{{\prime\:}}\left(\eta\:\right)\:$and $\:{g}^{{\prime\:}}\left(\eta\:\right)$ when * M=0.4,* $\:\beta\:=1.2,$ $\:\alpha\:=0.5,$ $\:B=0.1,\:\beta\:=1.2,$ *Nr=1.5,* $\:{\theta\:}_{w}=1.1,$ and $\:{Ec}_{y}={Ec}_{x}=0.5.$.

The consideration of fluid motion in a Forchheimer porous medium results in a resistive force that opposes the flow. Two parameters $\:\lambda\:$ and * Fr* are raised due to the porous medium. The parameter $\:\lambda\:$ is due to the Darcy porous medium, whereas * Fr* it is due to the Forchheimer porous medium. Thus, the impact of Darcy and Forchheimer porous mediums on fluid is depicted in Figs. 13(a, b) and 14(a, b) respectively. These figures show that fluid flow experiences retardation due to the porous medium. It is found from simulations that $\:F{e}_{3}{O}_{4}$-* Co*-* Ni*-Casson nanofluid faces the highest retardation relative to mono- and di-nanofluids.

### Discussion on the temperature field under the variation of parameters

**Fig. 15:**
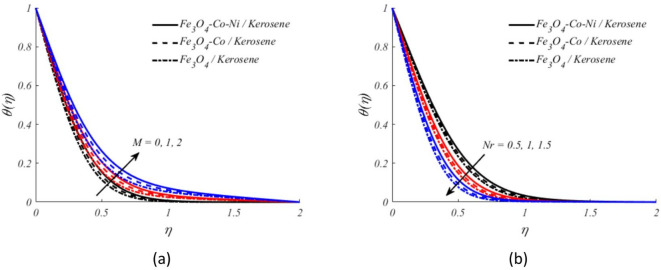
**(a**,** b)** Impact of Hartman number $\:M\:$and radiation parameter * Nr* on $\:\theta\:\left(\eta\:\right)$ when $\:\lambda\:=1.2,\:$ $\:\beta\:=1.2,$ $\:\alpha\:=0.5,$ $\:B=0.1,\:$ F* r=0.1,* $\:{\theta\:}_{w}=1.1,$ $\:{Ec}_{x}=0.1\:$and $\:{Ec}_{y}=0.1$.

**Fig. 16:**
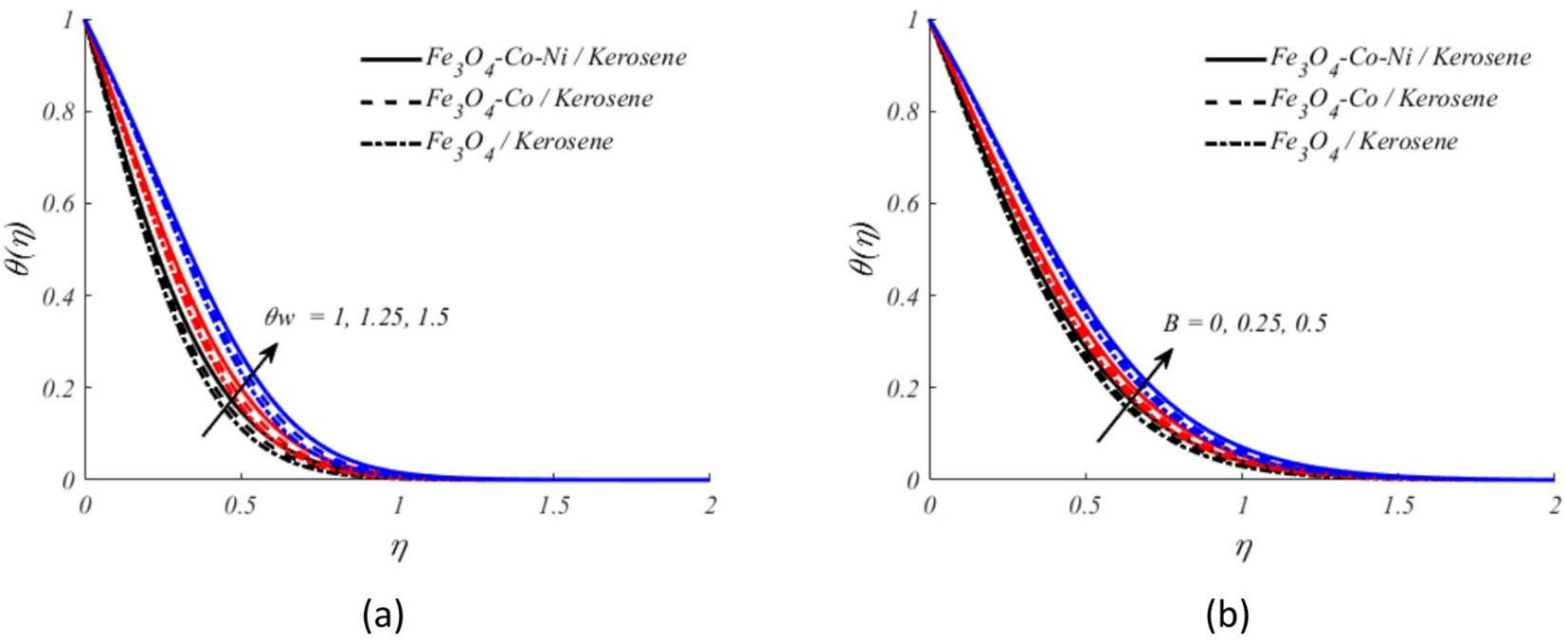
**(a**,** b)** Impact of temperature ratio parameter $\:{\theta\:}_{w}\:$and heat coefficient * B* on $\:\theta\:\left(\eta\:\right)$ when * M=0.1,* $\:\lambda\:=1.2,\:$ $\:\beta\:=1.2,$ $\:\alpha\:=0.5,$ $\:Nr=0.5,\:$ F* r=0.1,* $\:{Ec}_{x}=0.1\:$and $\:{Ec}_{y}=0.1$.

**Fig. 17:**
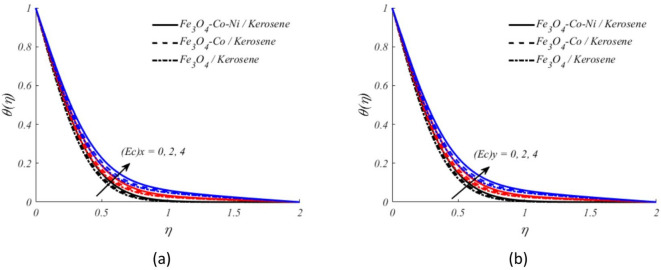
**(a**,** b)** Impact of Eckert number $\:{Ec}_{x}\:$and $\:{Ec}_{y}$ on $\:\theta\:\left(\eta\:\right)$ when * M=0.1,* $\:\lambda\:=1.2,\:$ $\:\beta\:=1.2,$ $\:\alpha\:=0.5,$ $\:Nr=0.5,\:$ F* r=0.1,* $\:B=0.1\:$and $\:{\theta\:}_{w}=1.1.$.

The dimensionless parameters (* M*, * Nr*, $\:{\theta\:}_{w}$, * B*, $\:{\left(Ec\right)}_{x}$ and $\:({Ec)}_{y}$ are raised as a result of nondimensionalization of energy equations and describe the impact of magnetic field, thermal radiation, temperature difference (between wall and ambient temperatures), heat generation, and ohmic dissipation, respectively. Numerical simulations, because of the change in aforementioned parameters, are executed, visualized, and recorded in graphical data displayed in Figs. 15(a, b)- 17(a, b). Figure 7a analyses the influence of an increase in the intensity of the magnetic field. The fact revealed by the simulations displayed in Fig. 15a predicts that the process of ohmic dissipation speeds up as the intensity of the magnetic field is increased. The process of ohmic dissipation becomes the cause of the increase in the conversion of electrical energy into heat energy, which has become the cause of an increase in temperature. Figure 15a also reveals that the ternary nanofluid converts electrical energy into heat higher rate than the conversion of electrical energy by $\:F{e}_{3}{O}_{4}$-* Co*-kerosene oil and $\:F{e}_{3}{O}_{4}$-Kerosene oil. As a consequence, the thermal region becomes wider due to heat dissipated by Joule heating. The ohmic dissipation is the phenomenon by which electric current experiences resistance when it flows through an electrically conducting material, and this resistance causes heating of the material through which the electrical current passes. Further, ohmic dissipation is considered in this study, and therefore the term $\:\frac{{\sigma\:}_{tnf}{B}_{0}^{2}}{{\rho\:}_{tnf}}\left({u}^{2}+{v}^{2}\right)$ in Eq. $\:\left(5\right)$ or its dimensionless form $\:Pr{M}^{2}\left(E{c}_{x}{{f}^{{\prime\:}}}^{2}+E{c}_{y}{g}^{{\prime\:}2}\right)$ in Eq. $\:\left(10\right)$ is due to the consideration of ohmic dissipation and shows that heat dissipated due to the ohmic dissipation is proportional to the electrical conductivity of the fluid i.e., heat dissipated is higher for fluids with higher thermal conductivity. Since effective electrical conductivity increases due to the dispersion of nanoparticles. Therefore, the highest increase in electrical conductivity is due to the dispersion of tri-nanoparticles. Thus, the highest electrical energy is converted into heat as shown in Fig. 17(a, b).

Figure 15b predicts the influence of thermal radiation on the temperature of $\:F{e}_{3}{O}_{4}$-* Co*-* Ni*-Kerosene oil. Since thermal radiations emitted by the fluid more away from the flow region, therefore, radiation while leaving the fluid carry heat with it because of which fluid’s temperature of fluid becomes lower. This decrease in temperature is obvious by Fig. 6b. Figure 15b also predicts that mono-nanofluid ($\:F{e}_{3}{O}_{4}$-kerosene oil) emits thermal radiations higher than radiations emitted by $\:F{e}_{3}{O}_{4}$-* Co*-* Ni*-kerosene oil and $\:F{e}_{3}{O}_{4}$-* Co*-kerosene oil. Obviously, thermal region becomes shorter due to thermal radiation process. Figure 16a analysis the impact of wall temperature on fluid whereas Fig. 16b predicts the impact of heat generation on temperature of fluid. Both wall temperature and heat generation enhance the temperature of fluids. Figure 16b has revealed that in $\:F{e}_{3}{O}_{4}$-* Co*-* Ni*-kerosene oil, the heat generation process is higher relative to other considered fluids. It is also important to point out that $\:{\left(Ec\right)}_{x}$ and $\:({Ec)}_{y}$ are parameters called Eckert numbers and coefficients of terms (of dimensionless energy equation) raised because of consideration of Joule heating and therefore, has an increasing impact on the temperature of all three types of nanofluids (see Figs. 17(a, b)).

## Final findings

A numerical investigation is performed to analyze the improvement in thermal performance of Casson fluid due to the dispersion of multi-nanoscale particles in the presence of Joule heating, thermal radiation, heat generation or absorption, and magnetic field. The animated results have depicted the following outcomes:

- The resistance of the porous medium to the flow of $\:\:F{e}_{3}{O}_{4}$-* Co*-* Ni*-Kerosene oil is more than that of mono- and di-nanofluids. Therefore, of $\:\:F{e}_{3}{O}_{4}$-* Co*-* Ni*-Kerosene oil exerts the highest drag on the surface of a solid boundary. Thus, fluid in a porous medium may damage the surface over which the fluid flows is the porous medium.
- The highest opposing Lorentz force is noted in the flow of $\:F{e}_{3}{O}_{4}$-* Co*-* Ni*-Kerosene fluid relative to di- and tri-nanofluids.
- It is established that $\:F{e}_{3}{O}_{4}$-* Co*-* Ni*-kerosene oil, $\:F{e}_{3}{O}_{4}$-* Co*-kerosene oil and $\:F{e}_{3}{O}_{4}$-kerosene oil, $\:F{e}_{3}{O}_{4}$-* Co*-* Ni*-Kerosene oil is the best working fluid concerning heat transport.
- The working fluid should be non-heat-generating and non-Ohmic dissipative. Further, a magnetic field should not be applied to avoid ohmic dissipation in fluids. Thus, in practical applications where fluid is used for heat transport, the fluid should not be exposed to a magnetic field, and the fluid should not be heat-generating.
- Porous medium creates a resistance to the flow, which reduces the convective heat transfer, and consequently, the heat transport rate decreases. Eventually, the local Nusselt number decreases.
- The yield stress is responsible for reducing the wall stress in both * x*- and * y*-directions. Thus, fluids with less yield stress have a wider boundary layer region than those exhibiting higher yield stress.c.
- The thermal radiation results in a decrease in temperature. Consequently, the temperature of the fluid decreases, and the thermal boundary layer thickness is reduced. Thermal radiation is also responsible for increasing the local Nusselt number.

### Future recommendations

In order to obtain a more accurate depiction of thermal transport phenomena, future research may expand the current model to include hybrid and ternary nanofluids, three-dimensional and unstable flow topologies, and additional physical effects. Prediction accuracy and computing efficiency can be further increased through the use of machine learning and optimization techniques, as well as experimental validation. The practical value of the current discovery would also be expanded by studies including various non-Newtonian fluid models, entropy generation analysis, complicated geometries, renewable energy, or biomedical applications.

## Data Availability

All data generated or analysed during this study are included in this published article.

## List of symbols

* u,v,w*: Velocity components $$\:\left(m{s}^{-1}\right)$$
$$\:\nu\:$$: Kinematic viscosity $$\:\left({m}^{2}{s}^{-1}\right)$$
$$\:{B}_{0}$$: Magnetic induction $$\:\left(Tesla\right)$$
$$\:\sigma\:$$: Electrical conductivity $$\:\left(S{m}^{-1}\right)$$
$$\:\rho\:$$: Density $$\:\left(kg{m}^{-3}\right)$$
$\:{k}_{p}$: Permeability of porous medium$\:\left({m}^{2}\right)$
* F*: Non-uniform inertial coefficient $\:\left({m}^{-1}\right)$
* T*: Temperature of fluid $\:\left(Kelvin\right)$
$$\:{T}_{\infty\:}$$: Ambient temperature $$\:\left(Kelvin\right)$$
$\:{\sigma\:}^{*}$: Stefan-Boltzmann constant
* Q*: Heat generation/absorption coefficient $\:(W\bullet\:{m}^{-3}\bullet\:Kelvin)$
$\:{k}^{*}$: Mean absorption coefficient $\:\left({m}^{-1}\right)$
$\:{c}_{p}$: Specific heat capacity
* K*: Thermal conductivity
$$\:{T}_{w}$$: Wall temperature $$\:\left(Kelvin\right)$$
* x,y,z*: Cartesian coordinates $$\:\left(m\right)$$
$$\:{V}_{w}$$: Wall velocity
$$\:{u}_{w}$$: Wall velocity $$\:\left(m{s}^{-1}\right)$$
$\:\beta\:$: Casson fluid parameter
$$\:{M}^{2}$$: Hartmann number
$\:\lambda\:$: Darcy porous parameter
* Fr*: Forchheimer number
* Pr*: Prandtl number
$$\:\alpha\:$$: Ratio parameter
* B*: Heat generation or absorption parameter
* Nr*: Radiation parameter
$$\:E{c}_{x}$$: Eckert number
$$\:E{c}_{y}$$: Eckert number
* Re*: Reynolds number
$\:{\theta\:}_{w}$: Temperature ratio parameter
* tnf*: Tri-nanofluid
$$\:{C}_{b}$$: Drag factor
$$\:{p}_{y}$$: Yield stress
* a,b*: Stretching constants
